# Boosting antitumor efficacy using docetaxel-loaded nanoplatforms: from cancer therapy to regenerative medicine approaches

**DOI:** 10.1186/s12967-024-05347-9

**Published:** 2024-05-30

**Authors:** Nima Beheshtizadeh, Zahra Amiri, Seyedeh Zoha Tabatabaei, Amir Abbas Seraji, Maliheh Gharibshahian, Akram Nadi, Morvarid Saeinasab, Farshid Sefat, Hanieh Kolahi Azar

**Affiliations:** 1 Department of Tissue Engineering, Faculty of Advanced Medical Sciences, Tabriz University of Medical Sciences, Tabriz, Iran; 2 Department of Materials Science and Engineering, Sharif University of Technology, 1458889694, Tehran, Iran; 3 Cardiogenetic Research Center, Rajaie Cardiovascular Medical and Research Institute, Iran University of Medical Sciences, Tehran, Iran; 4 Department of Mechanical and Industrial Engineering, University of Toronto, Toronto, Canada; 5 Department of Polymer Engineering and Color Technology, Amirkabir University of Technology, Tehran, Iran; 6 Department of Tissue Engineering and Applied Cell Sciences, School of Medicine, Semnan University of Medical Sciences, Semnan, Iran; 7 Stem Cell Biology Research Center, Yazd Reproductive Sciences Institute, Shahid Sadoughi University of Medical Sciences, Yazd, Iran; 8 Regenerative Medicine Group (REMED), Universal Scientific Education and Research Network (USERN), Tehran, Iran; 9 Department of Biomedical and Electronics Engineering, School of Engineering, University of Bradford, Bradford, UK; 10 Faculty of Biology, Medicine and Health, University of Manchester, Manchester, UK; 11 Interdisciplinary Research Centre in Polymer Science & Technology (Polymer IRC), University of Bradford, Bradford, UK; 12 Student Research Committee, Tabriz University of Medical Sciences, Tabriz, Iran; 13 Department of Pathology, Tabriz University of Medical Sciences, Tabriz, Iran

**Keywords:** Docetaxel-loaded nanoplatforms, Antitumor activity, Regenerative medicine, Tissue engineering

## Abstract

The intersection of nanotechnology and pharmacology has revolutionized the delivery and efficacy of chemotherapeutic agents, notably docetaxel, a key drug in cancer treatment. Traditionally limited by poor solubility and significant side effects, docetaxel’s therapeutic potential has been significantly enhanced through its incorporation into nanoplatforms, such as nanofibers and nanoparticles. This advancement offers targeted delivery, controlled release, and improved bioavailability, dramatically reducing systemic toxicity and enhancing patient outcomes. Nanofibers provide a versatile scaffold for the controlled release of docetaxel, utilizing techniques like electrospinning to tailor drug release profiles. Nanoparticles, on the other hand, enable precise drug delivery to tumor cells, minimizing damage to healthy tissues through sophisticated encapsulation methods such as nanoprecipitation and emulsion. These nanotechnologies not only improve the pharmacokinetic properties of docetaxel but also open new avenues in regenerative medicine by facilitating targeted therapy and cellular regeneration. This narrative review highlights the transformative impact of docetaxel-loaded nanoplatforms in oncology and beyond, showcasing the potential of nanotechnology to overcome the limitations of traditional chemotherapy and pave the way for future innovations in drug delivery and regenerative therapies. Through these advancements, nanotechnology promises a new era of precision medicine, enhancing the efficacy of cancer treatments while minimizing adverse effects.

**Graphical Abstract:**

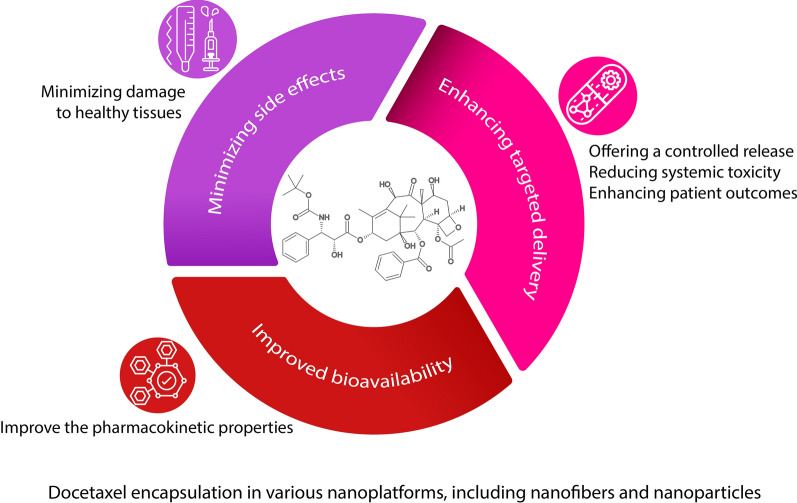

## Introduction

Cancer remains a major health issue worldwide, and its growing statics is worrying. Latest studies showed that age-standardized rates (ASRs) stand at 123.8/100,000 for men (a decrease of 6.5% compared to 2018) and 79.3 for women (a decrease of 3.7% compared to 2018) [1, 2]. On the other hand, current cancer treatments, including chemotherapy, immunotherapy, surgical tumor removal, and radiotherapy, often fall short of effectiveness due to absorption by healthy tissues, significant side effects, and inadequate absorption and penetration into the tumor, with only 2–5% of the therapeutic agent actually reaching the tumor site [3]. However, in clinical settings, chemotherapy agents remain a viable option for treating primary tumors and metastases. This underscores the necessity of developing a platform that can prolong and enhance their effectiveness, increase specificity, and reduce their side effects [4, 5].

Docetaxel (DTX), generating around $3.5 billion in revenue, is recognized as a leading chemotherapeutic drug. Its mechanism involves attaching to β-tubulin and interfering with the microtubules crucial for cell division and growth phases, effectively halting cancer cell proliferation and triggering apoptosis. [6, 7]. The worldwide market for DTX is anticipated to expand at an annual growth rate of 3.10% from 2023 to 2031, with Europe experiencing the most significant increase in this global sector. DTX is a new member of the taxane family, which is obtained from the precursor 10-deacetylbaccarin III from the European yew tree, and has better availability, water solubility, and effectiveness than the old member of the taxane family (Paclitaxel) [8, 9]. This drug was introduced in 2004 under the brand name Taxotere in America (Aventis pharmaceutical company) [10].

DTX is employed in managing various types of cancers, including prostate [11], lung [12], gastric adenocarcinoma [13], and breast [14], but it has limitations such as high hydrophobicity, containing a high concentration of ethanol and tween in its commercial formulation, rapid elimination, low selective distribution, and non-specific toxicities which reduced the efficiency of this chemotherapy agent [15, 16]. DTX treatment has led to significant adverse effects, including neutropenia, toxicity in the muscles and bones, allergic reactions, issues within the gastrointestinal tract, narrowing of the tear ducts, and toxicity affecting the skin [17]. In addition, the diverse pharmacokinetics of DTX has caused the variability of its toxicity and effectiveness and caused hematological toxicities [18].

In recent decades, the use of nanotechnology in drug delivery has attracted unprecedented attention [19]. Regarding cancer treatment, the conventional methods of delivering anticancer compounds face several obstacles, such as the tumor microenvironment, the mononuclear phagocyte system, and removal from blood vessels, while nanocarriers improve drug solubility and permeability, prevent drug degradation in the tumor microenvironment, and increase drug accumulation and bioavailability at the tumor site [20, 21]. Among these nanoplatforms, we can mention nanoparticles [22], nano vectors [23], nanofibers [24], liposomes [25], etc. as a method of administering DTX to enhance its effectiveness. Due to their diminutive scale and substantial surface area relative to volume [26], these nanoplatforms are capable of diminishing toxicity, regulating drug release, curtailing multidrug resistance, shielding the drug from immunological detection, and enhancing both the penetrative capacity and circulatory persistence of DTX [27].

Nanoplatforms reduce the side effects and dose of DTX and increase its half-life in clinical trials by selectively delivering the drug to the target tissue or cell [28]. Each nano platform has a different architecture, shape, and chemical composition that gives them unique properties [29]. These nanocarriers delivery is done in both active and passive ways [30]. However, the transfer of these approaches to the bedside has always faced extensive challenges [31].

**Fig. 1:**
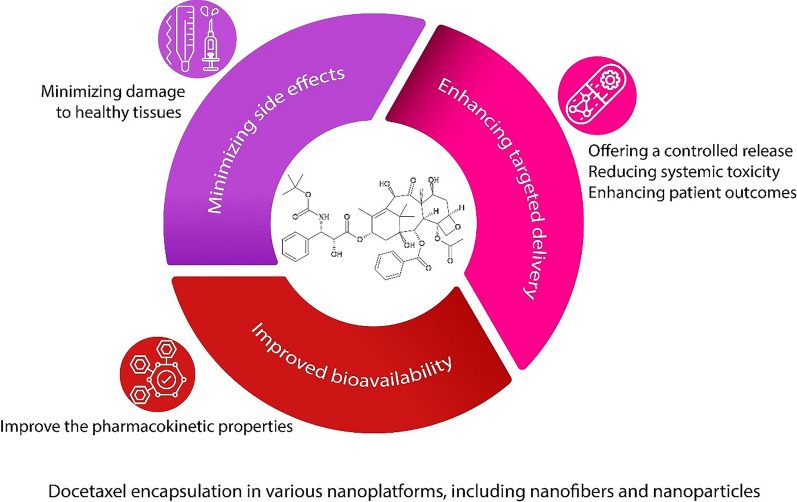
A schematic diagram of encapsulation of DTX in nanoplatforms, including nanoparticles and nanofibers, and their advantages

This study embarks on a comprehensive exploration of DTX, a prominent chemotherapeutic agent, within the scope of nanotechnology-enhanced medical treatments. Initially, we elucidate the molecular structure of DTX, inquire into its derivation and sourcing, and articulate its mechanism of action alongside its bioavailability. The crux of this review centers on DTX-loaded nanoplatforms, assessing the variety of nanosystems utilized for carrying docetaxel, their encapsulation efficiencies, and their therapeutic applications, particularly spotlighting the strides made in regenerative medicine. Figure 1 shows the advantages of DTX encapsulation in nanoplatforms.

We investigate the cutting-edge applications of these nanoparticles, scrutinizing the methodologies for encapsulating DTX at the nanoscale and the subsequent biological and physical impacts. The review aims to evaluate different nanoplatforms used as carriers for DTX, highlighting their efficacy in minimizing side effects and enhancing targeted delivery to tumor sites. As we conclude, we project into the future of DTX applications, signaling the potential shifts and advancements anticipated in this dynamic field.

## Docetaxel

### Structure, source, and derivate

DTX (807.89 Da), with a molecular weight of 807.89 Da, is a tetracyclic diterpenoid crystalline powder. Its chemical formula is C_43_H_53_NO_14_·3H_2_O. This compound features a tert-butyl carbamate ester and a hydroxyl group in the phenylpropionate side chain at carbon 10, respectively [32]. Tert-butyl carbamate ester has an essential role in antineoplastic activity. DTX is a hydrated (with 3 water molecules per drug molecule) and stable compound under ambient conditions. This white, fat-soluble medication melts at 232 °C, exhibits a membrane permeability rate of 1 × 10^–6^ cm/s, and possesses a water solubility of 0.025 μg/ml.[33].

In the 1980s, a French scientist named Pierre Potier synthesized DTX from a natural precursor called 10-deacetylbaccarin III obtained from the needles of the European yew tree, Taxus baccata, and with the help of the French pharmaceutical company Rhône-Poulenc and Université de Grenoble, it was advanced to the stage commercial production [34–36]. The promising function of this anticancer medication in managing diverse cancers has prompted the creation of several analogs to enhance its efficacy [37].

### Mechanism and bioavailability

DTX toxicity depends on the route of administration and its pharmacokinetic profile [33]. It has three different phases with different half-lives in its pharmacokinetic profile: alpha (4.5 min), beta (38.3 min), and gamma (12.2 h) [38]. DTX is typically administered through intravenous injection (weekly or every three weeks), leading to elevated levels of the medication in the liver, muscles, stomach, and bile ducts, although its clearance also occurs quickly and in the first 15 min [38]. After intravenous injection, the high expression of acid glycoprotein α-1 (AAG) in cancer patients and the ability to bind DTX to AAG has led to the drug binding to cancer cells more than healthy cells.

DTX predominantly binds to lipoproteins, albumin, and other plasma proteins [39]. It distributes from the body’s core to its extremities at a rate of 22 L/h/m^2^ [33]. Nonetheless, the significant concentration of P glycoprotein in the gastrointestinal tract and DTX’s strong tendency to bind to it result in an oral bioavailability of less than 10% when the drug is consumed by mouth.[40]. DTX metabolism is generally done in the liver and excreted through the kidneys, intestines, and bile [38].

DTX exerts its antitumor activity through different pathways. Initially, it interrupts the cell cycle at the G2/M phase, triggering cell death, inhibits the activation of anti-apoptotic genes Bcl-2 and Bcl-xL, and boosts the production of the cell cycle inhibitor p27 [41]. Microtubules are essential cytoskeleton components in cancer cell division, signaling, migration, and metastasis [42]. Microtubules are formed by non-covalent bonding of tubulin heterodimers. DTX binds to free tubulin causing its assembly into stable microtubules and preventing disassembly, so stabilizes microtubule bundles lacking normal function and prevents the division of cancer cells. DTX’s twofold binding affinity to tubulin compared to paclitaxel is one of the main reasons for its improved performance [17, 43].

## Docetaxel-loaded nanoplatforms

### Nanofibers

Nanofibers (NFs) have garnered significant interest as drug delivery vehicles because of their characteristics, including an elevated ratio of surface area to volume, excellent porosity, good capacity for loading drugs, flexibility, easy fabrication, and their capacity to be produced from various polymers. Due to a similar structure to the ECM, NFs could improve cell behaviors for biomedical usage [44, 45]. Local cancer therapy, drug administration, tissue healing, pH-sensitive therapy, gene delivery, and stent coating are just a few of the multiple prospective applications for these NFs [46, 47]. The application of NFs for administering DTX in cancer treatment, along with the ways in which this medication can be combined with nanofibers, will be discussed in the following sections.

#### Applications of docetaxel-loaded nanofibers

The most common cancer treatment is chemotherapeutic drugs and their combinations, while a significant issue with chemotherapy is the harm its side effects cause to healthy cells. The potential for high-quality, safe, and effective chemotherapy in a wide range of cancer types can be generated via localized drug delivery [21, 45]. Electrospun NFs can improve the administration of drug delivery methods. Most notably, they increase the drug’s ability to dissolve, making it more stable and effective in vivo [48].

DTX, a chemotherapeutic agent, plays a crucial role in treating various cancers by binding to tubulin. This binding stabilizes microtubules, diminishing their dynamic behavior. Consequently, it induces mitotic arrest, resulting in apoptosis across a wide array of tumor cells at the molecular level [49]. Because of its weak solubility and significant toxic side effects, DTX has limited clinical application. Using a delivery vehicle, like electrospun NFs, can help overcome DTX’s drawbacks and make it useful for treating local recurrences of cancer [50–52].

In a previous study, NFs infused with DTX and made of poly (d, l-lactide) (PDLLA) were utilized to stop the spread of breast cancer in earlier studies [53]. Findings from experiments conducted both in vitro and in vivo revealed an increase in apoptosis in 4T1 cells and demonstrated the biocompatibility of NFs following their implantation at the target site. It was seen that these structures could be useful for local chemotherapy [53]. In another study, the effects of DTX and lentinan, a natural substance, loaded in polyvinyl alcohol (PVA) nanofiber was investigated on breast cancer and results showed a notable decrease in the survival of MCF-7 cells and in the expression levels of the HER3 gene [24].

The use of DTX in carbon nanomaterials can be useful in improving prostate cancer by minimizing the dosage, systemic adverse effects, and chemoresistance through local delivery of chemotherapeutic medications [54, 55]. Researchers utilized biodegradable polymers made of polylactic acid (PLA)-polyethylene oxide (PEO)-poly (phenylene oxide) (PPO)-PEO-PLA, created self-assembled nanofibrous microspheres that co-encapsulate the drugs DTX and curcumin [56, 57]. In vitro findings demonstrated a continuous release of both encapsulated drugs, potentially aiding animal models in maintaining localized drug concentrations*.* Synergistic anticancer effects from the DTX and curcumin co-loaded NFs were observed in CT26 tumor in vivo, based on their report [57]. Also, the transmucosal delivery of DTX via PVA nanofibers was effectively accomplished in another previous study [58]. The results indicated a notable enhancement in mucoadhesive capabilities and a the delivery system led to a diminished survival rate of cancer cells within it [58]. The summary of studies on the use of DTX in NFs is listed in Table 1.

**Table 1:** DTX delivery for cancer therapy

No	Material and Study model	Cell type	Target site	Encapsulation method	Key results	Conclusion	Refs.
1	PDLLA nanofiber-DTX-mice	4T1 cell line	Breast cancer	Emulsion	In 4T1 cells, DTX /PDLLA nanofibers induced apoptosis. Biocompatibility of DTX/PDLLA nanofibers was observed after implantation in target site	Nanofibers composed of DTX and PDLLA may have tremendous potential for clinical applications requiring local chemotherapy	[53]
2	CNFs and CNTs-DTX-mitomycin C-in-vitro	DU-145 PCa cells	Prostate cancer	Cells were treated with drugs	reduced the viability of PCa cells that was more in the CNFs group and anti-tumor effects was observed	Carbon nanomaterials could reduce dosage, systemic adverse effects, and chemoresistance by local delivery of chemotherapeutics	[55]
3	PLA-PEO-PPO-PEO-PLA-DTX-mice	CT26 cells and L929 cells	Colon Cancer	Emulsion	Releasing of drugs slowly, increased apoptosis of tumor cells, and inhibited angiogenesis, and control of Cancer in the mice Colon	The microspheres, loaded with two drugs and made of nanofibers, hold promising potential for treating abdominal metastases in colorectal cancer	[57]
4	PGCL/PLGA-DTX, cabazitaxel-in-vitro	PC-3 and DU145 cells	Prostate cancer	Blending	Burst release of DTX than cabazitaxel and PGCL/PLGA + CTX was selected for anticancer analysis; decreased cell growth, good biocompatibility	Bioresorbable patches filled with cabazitaxel show promise as a drug delivery device for the treatment of prostate cancer	[59]
5	PVA-DTX* -*in-vitro	T47D cells	Oral cancer	Blending	Decreasing cell viability in the carrier system compared to the control group	Polymeric nanofibers can deliver anticancer drugs locally	[58]
6	Polycaprolactone-chitosan-DTX or doxorubicin-in-vitro	MCF-7 and T47-D	Breast cancer	Cells were treated with DTX	Increasing in markers related to mammary stem cell, sphere formation, inhibit differentiation of BCSC, rise in DTX-doxorubicin resistance	These scaffolds could be a good model for observing BCSC and how they react to anticancer drugs	[60]
7	Collagen-DTX and camptothecin*-*in-vitro	C4-2B Cells	Prostate cancer	Treating after cell culture	Microfibrous membrane showed better tumor microenvironment than other groups, formation of colonies like tumors, electrospun scaffolds indicated, cells on electrospun scaffolds indicated more resistance to both chemotherapy agents than other groups	This framework provides a controlled and reliable cell culture model useful for studies in cancer research and regenerative medicine	[61]
8	PCL/ZnO*-*DTX*-*in-vitro	Lung cancer cell line (A549)	Lung cancer	Blending	Constructs containing nanofibers and DTX indicated minimum toxicity to natural cells, promoted apoptosis of cancer cells	PCL + ZnO + DTX nanofibers can act as a targeted delivery method for the treatment of recurrence lung cancer	[48]
9	PDO/gelatin DTX, Cisplatin and Fluorouracil-mice	CD24 + and CD44 + cancer stem cells	In-vitro and in-vivo-Gastric cancer	Adding after implantation	In vitro and in vivo carcinogenesis increased without apoptosis	Depleting CD24 + and CD44 + cells made these medicines efficient stomach tumor treatment	[62]
10	PVA-lentinan and DTX-in-vitro	MCF-7 cells	Breast cancer	Blending	Breast cancer cell viability and the HER3 gene expression reduced in PVA/lentinan/DTX group than other groups	The use of lentinan, a natural substance, with a common chemical anticancer medicine and nano-drug delivery technology may be a viable cancer treatment	[24]

Breast-cancer stem-like cells (BCSC), Poly Lactic-co-Glycolic Acid (PLGA), Poly(ethyleneoxide)–poly(propylene oxide)–poly(ethylene oxide) (PEO–PPO–PEO), Poly(glycolide-"-caprolactone) (PGCL), Poly(lactic acid) (PLA), Polycaprolactone (PCL), Polydioxanone (PDO), Polyvinyl alcohol (PVA), Zinc oxide (ZnO)

#### Techniques used for encapsulating docetaxel in nanofibers

Drug delivery systems play a pivotal role in therapeutic application. Conventional drug delivery systems often fall short of achieving the desired therapeutic effect due to inadequate drug distribution at the target site and rapid drug elimination from the body, necessitating frequent injection dosages. The loading strategy is selected in accordance with the properties and function of the drug-incorporated fibers [63]. Direct blending of the drug and polymer solution, coaxial electrospinning, surface immobilization after spinning, and emulsions can be used to introduce drugs into fibers and each approach releases drugs differently [64].

##### Blend electrospinning

**Fig. 2:**
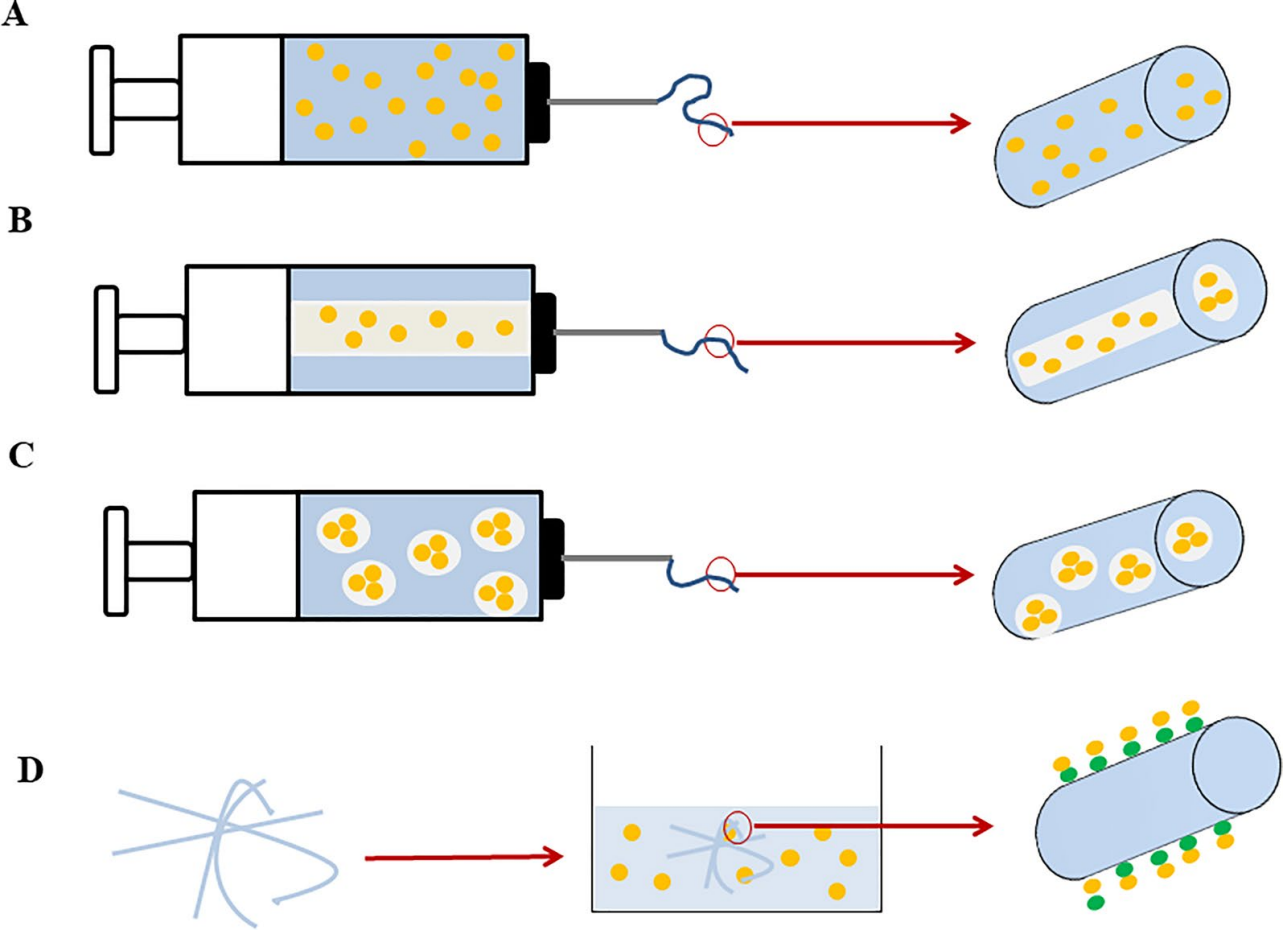
Schematic illustration of drug loaded Electrospun fibers. **A** Blend electrospinning involves co-solving pharmaceuticals and polymers in solutions before spinning, **B** dual concentric nozzles are used in co-axial electrospinning to spin distinct medicament and polymer mixtures, **C** Emulsion electrospinning involves the emulsification of medication solutions into insoluble polymer solutions, which is subsequently followed by the spinning process, **D** Following the immobilization process, drugs are attached to artificially created nanofiber matrices through either physical or chemical interactions

In the blending electrospinning method, drug molecules within the solution, including pharmaceuticals, undergo dissolution or dispersion, resulting in the formation of encapsulated drug entities through a one-phase electrospinning process, contingent upon their solubility characteristics. This step is executed prior to commencing the electrospinning procedure. Although this approach can be readily implemented, it does come with some associated limitations. When organic solvents come into direct contact with sensitive molecules like proteins, it is possible that the proteins will become denatured and lose their function. Since a large proportion of bioactive molecules carry electric charges, one limitation of the blending method is the migration of these molecules towards the jet’s surface during the process. Consequently, the fibers exhibit a higher concentration of bioactive molecules on their surface, as opposed to a uniform distribution throughout the structure (Fig. 2A) [65, 66].

##### Coaxial electrospinning

The coaxial technique is a modified form of electrospinning that pumps two distinct solutions via the nozzles (it is included two nozzles in a concentric arrangement) to manufacture nanofibers with core/shell architecture. Both solutions are maintained separate until the last moment to ensure that they do not have any opportunity to contact each other. During the process of coaxial electrospinning, the solution containing the biomolecules (such as: DNA, RNA and drugs) creates the inner jet, which results in the biomolecules receiving more protection, and another solution, which forms the outer jet, is co-electrospun during the process (Fig. 2B) [67].

When an electrospun nanofiber needs to carry drug that could be inactivated in vivo before it can carry out its intended function, a core/shell arrangement is a viable option, and the target molecule is encased in a protective fiber shell. Core/shell polymers concentration, drug concentration and molecular weight and the relative flow rates of the drug's core and shell solutions are factors that influence the effectively encapsulated of drugs in the core of the co-electrospun fibers [68, 69].

This approach can be utilized for biomolecule encapsulation and medication delivery. It was also used in the development of tissue engineering scaffolds to provide successful local, efficient, and consistent growth factor and gene delivery to cells seeded on the scaffold. Nevertheless, it does have some drawbacks, such as the requirement of exact regulation of process parameters such as the surface tension and viscoelasticity of the two polymers, as well as the complexity of the design [65, 67].

##### Emulsion

In the process of emulsion electrospinning, the aqueous drug is combined with the polymer solution, also known as the oil phase. Following electrospinning, if a low-molecular-weight medication is used, the biomolecule-loaded phase can be evenly dispersed throughout the fibers, while the incorporation of macromolecules into the aqueous phase results in a core/shell fibrous structure. This method removes the necessity for a common solvent, allowing a wide variety of hydrophilic pharmaceuticals to be combined with hydrophobic polymer compounds, and minimized interaction between drugs and organic solvent during the electrospinning process. However, the shearing force and tension between the emulsion's two phases present a risk that delicate biomolecules like nucleic acids will be damaged or destroyed (Fig. 2C). Biomolecule distribution in fibers depends on the hydrophilic-to-hydrophobic solution ratio. This parameter controls the encapsulated drug’s releasing profile, durability, and bioactivity [63, 67, 68].

##### Immobilization

After the electrospinning process, the surfaces of fibers can be modified through either physical or chemical means to immobilize bioactive molecules, thereby providing specific biochemical signals to cells. By employing this method, therapeutic agents and pharmaceuticals are conjugated or bonded to the nanofiber surfaces, rendering the nanofibers biochemically and structurally akin to normal tissue (Fig. 2D) [66, 67].

In the chemical immobilization approach, the biomolecule forms a covalent link with the carrier, resulting in a gradual and controlled release of the biomolecule. Because of this, it is better suited for delivery of genes or growth factors in situations in which a slow and protracted distribution is necessary. Nevertheless, this method is not suitable for immobilizing medications that must undergo endocytosis or interact with the cell nucleus. Plasma treatment, a wet chemical technique, and surface scaffold polymerization are all forms of surface modification that can be applied to nanofibers [68, 70].

The simplest technique for embedding drugs into electrospun nanofibers involves physical adsorption onto their surface. This method relies on four main types of interactions: electrostatic, hydrogen bonding, hydrophobic, and Van der Waals. Within these mechanisms, the Van der Waals force is recognized as the simplest method for immobilization via physisorption. Where the high surface area to volume ratio enhances drug loading capacity. However, there is a risk of drug shedding in physisorption, stemming from weak adsorption between the drug and its carrier [68, 69].

### Nanoparticles

#### Applications of docetaxel-loaded nanoparticles

The administration of anticancer agents via systemic pathways and standard approaches faces numerous challenges due to tumor properties that resist conventional therapies. Conventional therapies often fall short in completely removing tumors because drugs must overcome biological barriers such as the mononuclear phagocyte system, the vascular endothelial layer, and the tumor’s microenvironment [71]. Thus, nanoparticle delivery systems could offer a promising alternative to traditional drug delivery mechanisms for transporting anticancer drugs like DTX [7]. Emerging as a new platform, these innovative drug delivery systems target breast cancer, non-small cell lung cancer (NSCLC), and prostate cancer [38]. These systems offer numerous benefits over traditional drug delivery methods, including controlled release of medication, precise targeting of tumors, and reduced overall toxicity compared to unbound DTX, as explored in the studies.

##### Breast cancer

In women, breast cancer ranks as the most prevalent type of malignant tumor, with the majority of cancer-related deaths stemming from metastatic spread [72]. Therefore, chemotherapy prevents recurrence and metastasis and prolongs the patient's life [73]. A key impact of DTX on breast cancer cells lies in its capacity to attach to β-tubulin subunit microtubules and inhibit their polymerization, thereby arresting cell growth during the G2-M phase [74]. Efforts to overcome the limitations associated with DTX are being made to develop an advanced tumor-targeting delivery system, which we will discuss below.

**Fig. 3:**
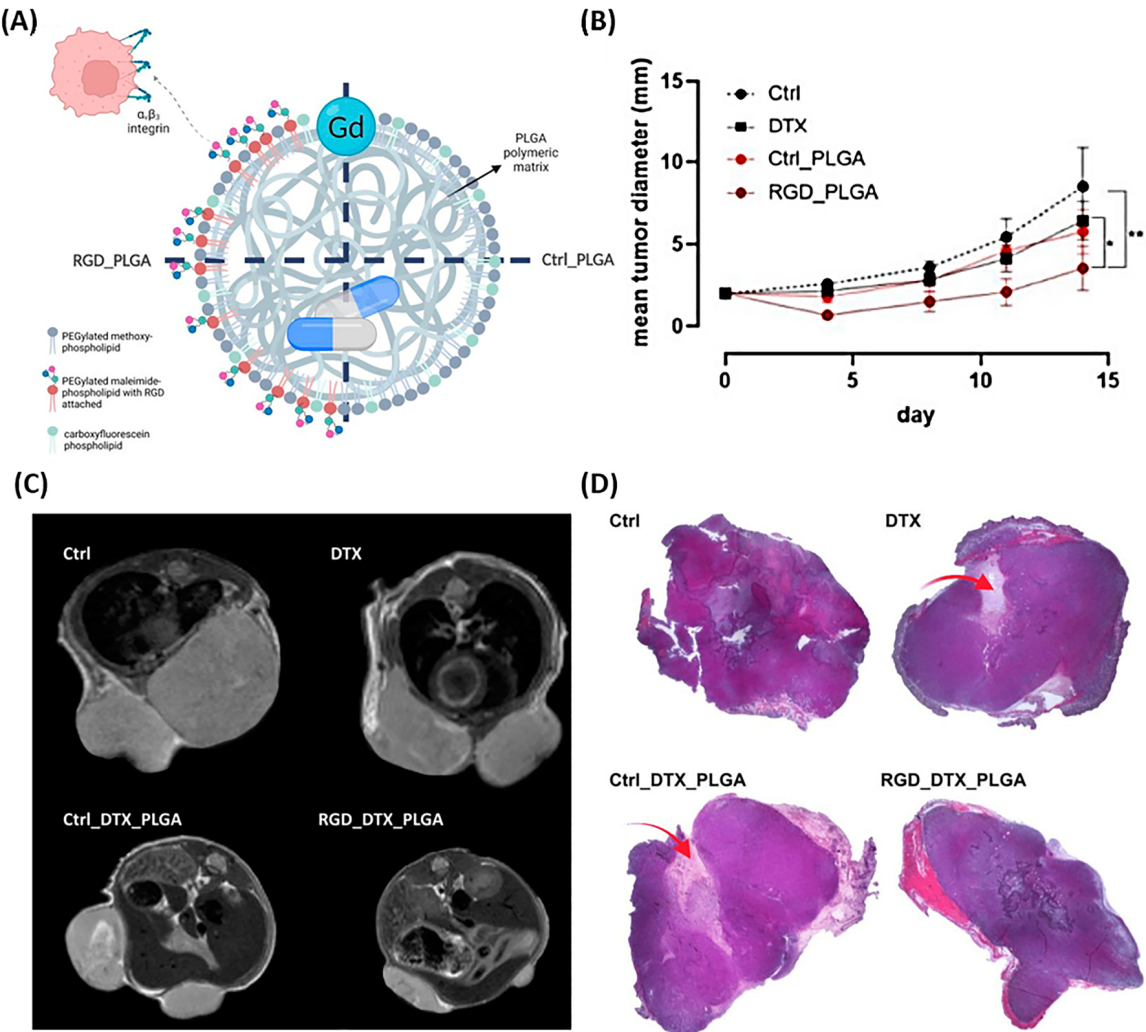
Depiction of RGD_PLGA Nanoparticles with DTX. **A** Schematic of PLGA-NPs. **B** In vivo effect of PLGA-NP on tumor diameter at the end of the experiment. *p < 0.05; **p < 0.01; two-way ANOVA. A study of the MRI and histological characteristics of 4T1 tumors obtained from mice. **C** Representative axial T_2w_-MR images of tumor region in mice with RGD_PLGA, Ctrl_ PLGA, free DTX, or physiological saline solution as a control (N = 6 per group). **D** Representative tumors from four treatment groups were histologically stained with hematoxylin/eosin. Necrotic areas are indicated by red arrows (Reprinted with permission from [77])

Gaikwad et al. [75] developed DTX-loaded niosomes to investigate the anticancer effect on breast cancer. The sustained release of DTX was successfully enhanced by niosomes, enabling gradual delivery of the medication to the targeted area while minimizing toxicity to surrounding cells. Hence, incorporating DTX into niosomes proves to be an efficient approach for enhancing its solubility, lowering its toxicity, and increasing the drug’s stability against cancer [75]. Carvalho et al. [76] designed a lipid nanostructure containing copaiba oil (CO) loaded with DTX (NLC_DTX_). The optimized formulation (NLC_DTX_) improved the encapsulation efficiency, increased the drug release time, and decreased the viability of breast cancer cells (4T1/MCF-7) compared to the commercial DTX. Therefore, considering the anticancer effects and the ability of stable drug release by NLC_DTX_, this method holds promise as an effective drug delivery system for breast cancer treatment [76]. Gregorio et al. [77] for breast cancer treatment, poly (lactic-co-glycolic acid) nanoparticles (PLGA-NPs) encapsulating DTX were created, featuring an Arg-Gly-Asp (RGD) tripeptide designed to specifically target αvβ3 integrins, which are abundantly expressed in breast cancer cells (Fig. 3A).

Observations highlighted that these NPs increased the antitumor effect and drug accumulation in the tumor, significantly disrupting tumor growth, according to Fig. 3B. Compared to free DTX, targeted PLGAs were used to successfully transport the drug to both triple-negative and HER2+ breast cancer cells in clinical models, halting tumor growth. Furthermore, encapsulating DTX in NPs minimized its accumulation in remote organs like the heart, thereby decreasing adverse effects (Fig. 3C) [77]. Research showed that mice receiving RGD_PLGA and Ctrl_PLGA treatments exhibited significant reductions in tumor size, attributed to the beneficial effects of PLGA. Moreover, RGD_PLGA treatment was associated with a reduction in necrotic regions within the mice (Fig. 3D) [77].

**Fig. 4:**
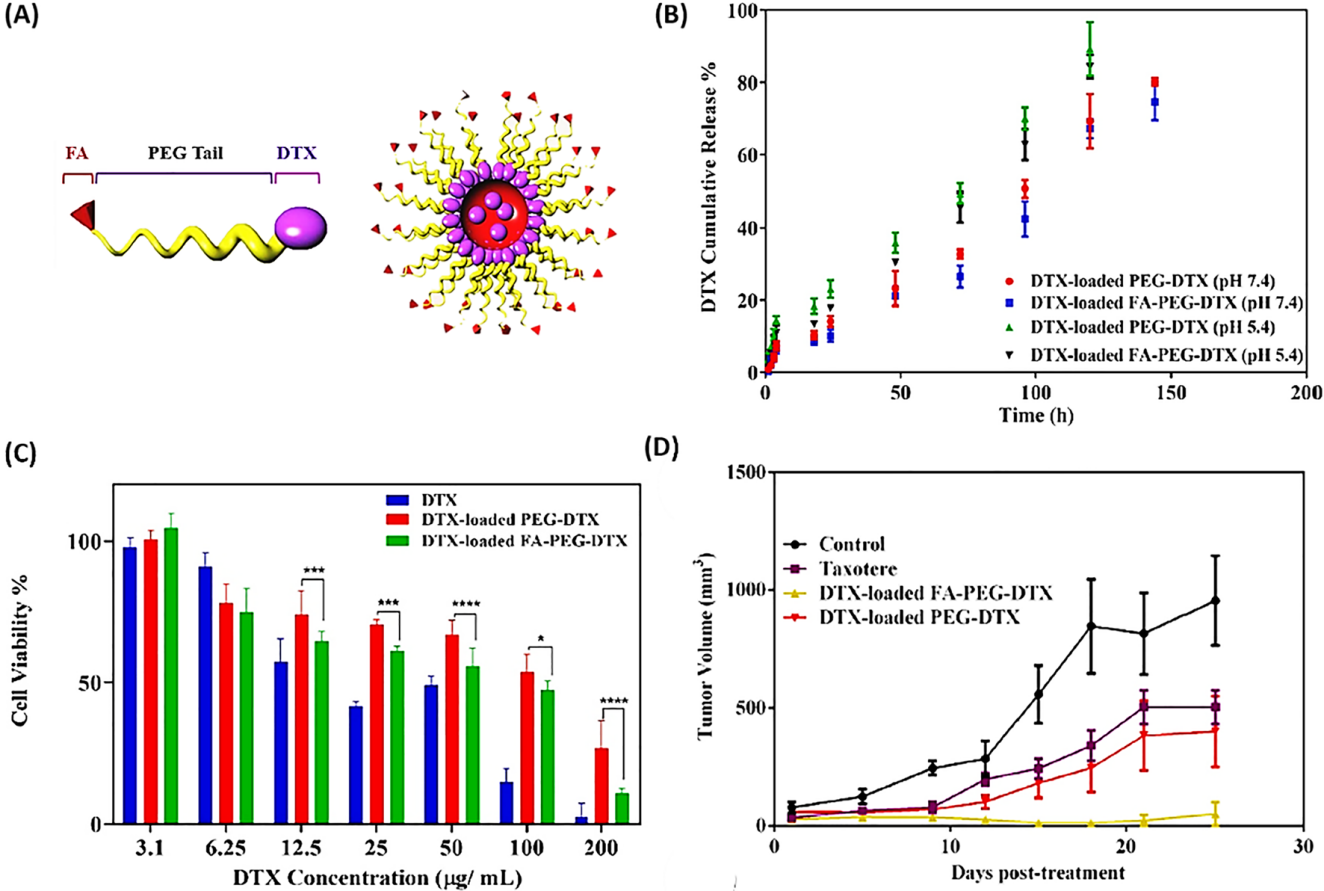
Folate-PEG-DTX on a Nanoscale. **A** Depicts the structure of the DTX-PEG micelle. **B** Illustrates the release behavior of DTX at 37 °C using DTX encapsulated in FA-PEG-DTX and PEG-DTX formulations in both citrate buffer (pH = 5.4) and phosphate buffer (pH = 7.4). **C** Shows the cytotoxic impact on the 4T1 cell line following 48 h of exposure to DTX encapsulated in FA-PEG-DTX, DTX encapsulated in PEG-DTX, and unencapsulated DTX. **D** Details the comparative in vivo efficacy of various formulations on tumor size reduction in BALB/c mice implanted with 4T1 tumors (n = 4) (Reprinted with permission [78])

Andisheh et al. [78] reported that a specialized micellar blend, containing DTX, folic acid (FA), and polyethylene glycol (PEG) was developed for targeting metastatic breast cancer 4T1 cells in both laboratory and live organism studies (Fig. 4A). Findings indicated that this drug delivery mechanism enhanced the capacity for DTX loading within micelles as well as facilitated a controlled and prolonged release of the drug (Fig. 4B). In addition, Fig. 4C and D demonstrate the impact of the FA-DTX-PEG complex loaded with DTX on cellular toxicity and antitumor effect compared to Taxotere against 4T1 cells [78].

Saqr et al. [79] reported that a novel formulation was developed using silk fibroin-based nanoparticles (SF-NPs) encapsulating DTX for the purpose of examining their cytotoxic and apoptotic effects on breast cancer cell lines. The outcomes demonstrated enhanced cytotoxic efficacy when DTX was encapsulated within SF-NPs, compared to its free form, along with increased uptake of DXL by MCF-7 and MDA-MB-231 cell lines. Furthermore, the internalization of SF-NPs led to a higher concentration of DXL in the G2/M phase, resulting in more effective cell cycle arrest. Overall, the encapsulation of DXL in SF-NPs not only amplified its cytotoxic impact by boosting drug absorption by cancer cells but also protected healthy cells from the adverse effects associated with the unencapsulated drug [79].

**Fig. 5:**
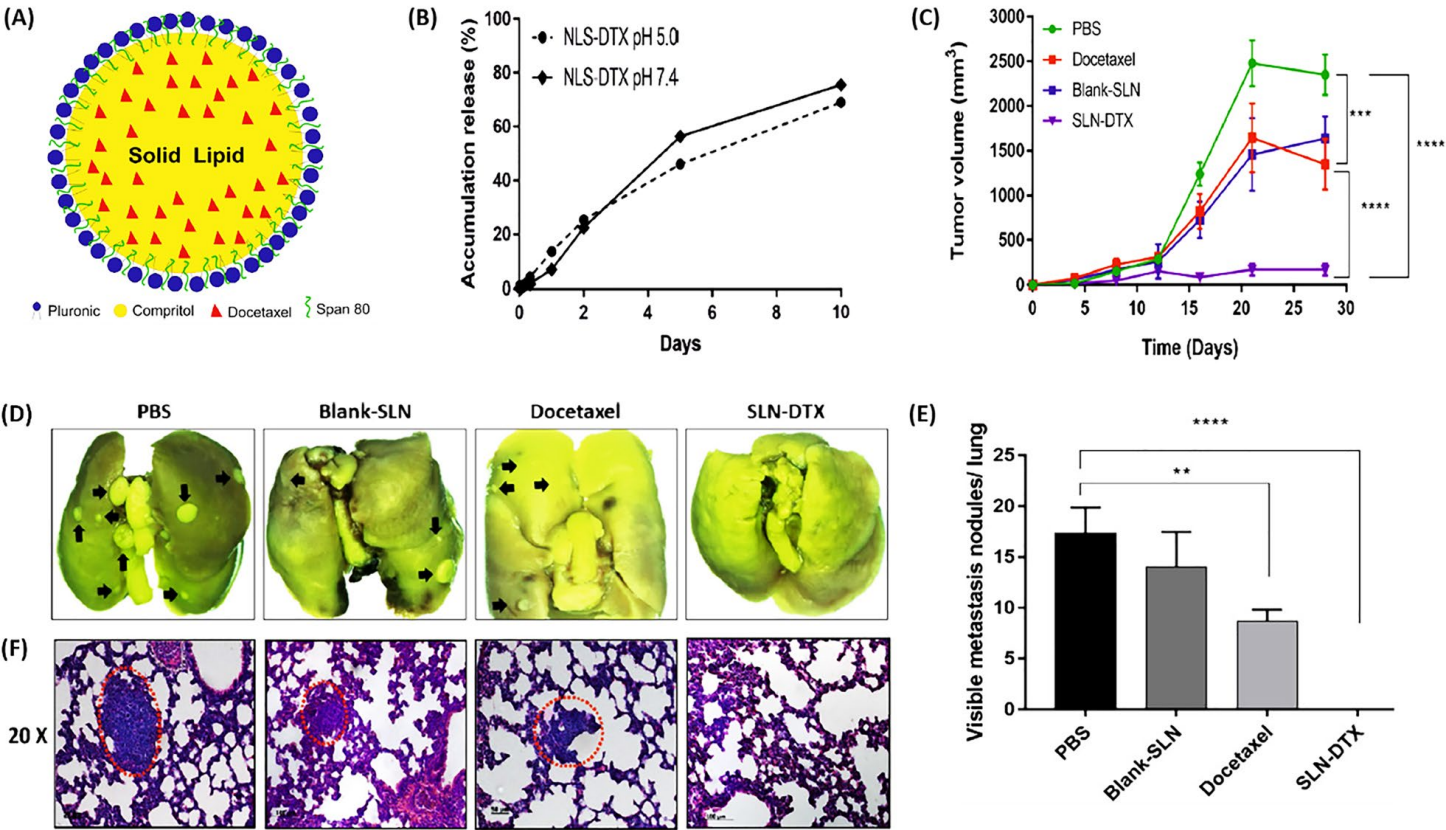
DTX-loaded solid lipid nanoparticles Solid lipid nanoparticles encapsulating DTX. **A** The composition of SLN-DTX. **B** Release pattern of SLN-DTX at neutral (pH 7.4) and acidic (pH 5.0) conditions in PBS over a period of 10 days, presented as average ± standard error (****p < 0.0001). **C** Changes in tumor size in Balb/c mice bearing 4T1 tumors treated with SLN-DTX, DTX, Blank-SLN, and PBS, with significant distinctions noted against the PBS group (***p < 0.001; ****p < 0.0001). **D** Lung image. **E** Statistical evaluation of lung tumor nodules. **F** Microscopic examination of lung tissue, highlighting metastatic locations with black arrows and red dotted lines. Averages ± standard error are provided (**p < 0.01; ****p < 0.0001) (Reprinted with permission from [80])

As depicted in Fig. 5A, Rocha et al. [80] developed solid lipid nanoparticles (SLNs) loaded with DTX (SLN-DTX) aimed at treating metastatic breast tumors in 4T1-bearing BALB/c mice. These SLNs achieved an 85% encapsulation efficiency for DTX, and controlled release of the drug was demonstrated in Fig. 5B. In vivo experiments indicated that SLN-DTX had a more pronounced antitumor effect by diminishing tumor size, as illustrated in Fig. 5C, compared to the administration of free DTX. Figure 5D and F reveal the absence of lung metastasis in mice treated with SLN-DTX, in contrast to the PBS and Blank-SLN groups, which exhibited tumor metastasis, and the DTX group, which showed a moderate level of metastasis. Thus, they engineered nanocarriers for DTX that offer enhanced therapeutic efficacy in the treatment of cancer [80].

Gaio et al*.* [81] reported that simultaneous delivery of DTX and the photosensitizer meso-tetraphenyl chlorine disulfonate (TPCS2a) was achieved using NPs coated with hyaluronic acid (HA-NPs) for the dual purpose of chemotherapy and photodynamic therapy (PDT) in treating breast cancer cells. Laboratory studies indicated that the efficacy of the combined therapy with nanoparticles containing both DTX and TPCS2a (HA@DTX/TPCS2a-NPs) surpassed that of therapies using NPs loaded with either DTX (HA@DTX-NPs) or TPCS2a (HA@TPCS2a-NPs) alone. Hence, authors reported that the combined delivery of chemotherapy agents and photosensitizers for PDT through specifically designed NPs, which target and eliminate cancer stem cells, enhances the success rate of cancer therapies [81]. Their claim is in parallel with other studies [82–84].

Also, Emami et al. [28] reported that a polymeric micelle responsive to pH changes was developed, incorporating alpha-tocopherol (TOC) and heparin (HEP), and was formulated with DTX for attacking breast cancer cells. The DTX-infused HEP-CA-TOC micelles demonstrated a more gradual drug release and enhanced stability in an acidic environment compared to neutral conditions. Consequently, the NPs carrying DTX were more effective in killing breast cancer cells (MCF-7 and 4T1) than the free form of DTX, attributed to greater cellular absorption and the release dynamics of the anticancer compound in the acidic milieu of the endosome [28].

Lu et al*.* [85] reported that methoxy poly(ethylene glycol 2000)-b-triacontanol (mPEG2k-b-TRIA) was utilized to create DTX-encapsulated polymeric micelles (DTX-PMs) aimed at treating breast cancer. These DTX-PMs enhanced the drug's exposure and prolonged its circulation time within tumor cells. Demonstrating superior anticancer efficacy, the DTX-PMs were more effective at suppressing the proliferation of breast cancer cells both in laboratory settings and live models (in MCF-7 cells and cancer-bearing BALB/c mice) compared to a solution of DTX [85].

Zafar et al. [86] designed a novel formulation combining DTX with thymoquinone (THQ) within mPEG-DSPE-Vitamin E TPGS-Lipid nanocapsules (DxTq-LNCs), which was crafted for breast cancer therapy. The DxTq-LNCs, featuring a controlled release mechanism and effective drug loading, enhanced resistance to protein binding and preserved the antioxidant properties of THQ. These nanocapsules also significantly altered the morphology of breast cancer cells, showing evidence of apoptosis, and curtailed metastatic progression by inhibiting cell movement. Integrating agents that counter multidrug resistance (such as vitamin E, TPGS, and THQ) into the nanoformulations amplified DTX’s anticancer capabilities, particularly against triple-negative, resistant breast cancer cells. Toxicity assessments indicated that DxTq-LNCs did not adversely affect blood chemistry or tissue histology, marking them as a promising approach for combinational chemotherapy in breast cancer treatment. [86].

**Fig. 6:**
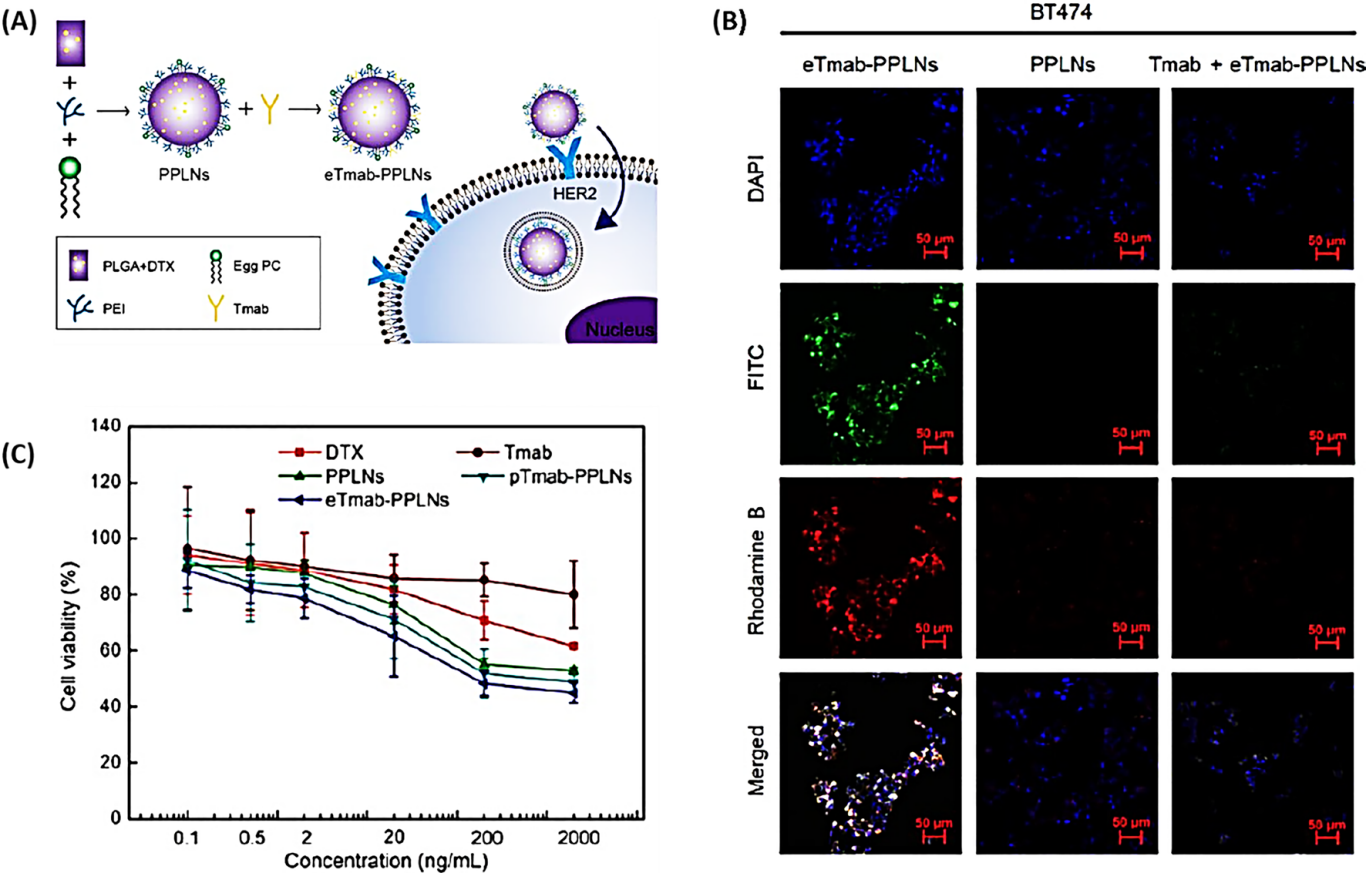
DTX encapsulated in trastuzumab-coated nanoparticles. **A** Depicts eTmab-PPLNs and their uptake by cells. **B** Demonstrates the cytotoxic effects on BT474 cells of various treatments: DTX solution, Tmab, PPLNs, pTmab-PPLNs, and eTmab-PPLNs. **C** Shows CLSM (Confocal Laser Scanning Microscopy) visuals of BT474 cells after incubation with fluorescently tagged PPLNs, eTmab-PPLNs, or a combination of Tmab and eTmab-PPLNs. Here, the mixture of eTmab-PPLN with Tmab is shown, where Tmab without fluorescence labels was introduced prior to the double fluorescence-tagged eTmab-PPLNs (Reprinted with permission from [87])

Zhang et al*.* [87] developed were trastuzumab (Tmab)-coated lipid-polymer hybrid NPs (PLNs), using PLGA to transport the anticancer drug DTX (Fig. 6A). These NPs are designed to hone in on breast cancer cells expressing high levels of human epidermal growth factor receptor 2 (HER2). Illustrated in Fig. 6B, the uptake of Tmab cells by HER2-positive cells was enhanced through HER2-mediated endocytosis using eTmab-PPLNs. Moreover, the fluorescence intensity observed in BT474 cells treated with Tmab + eTmab-PPLNs was markedly less than that seen with eTmab-PPLNs alone. Therefore, the in vitro studies on cellular uptake and cytotoxicity (Fig. 6C) indicate that these NPs are effective in delivering DTX to HER2-positive breast cancer cells, offering a promising approach for treating cancers characterized by elevated HER2 levels [87].

Jose et al*.* [88] conjugated transferrin (T*f*) with PLGA to load DTX trihydrate (DCT) into PLGA NPs for breast cancer treatment. Cell results showed that T*f*-conjugated PLGA NPs loaded with DCT were more active than their non-conjugated counterparts, had more cytotoxicity against MCF-7 cells, and had more effective anticancer activity by arresting the G2/M phase [88].

**Fig. 7:**
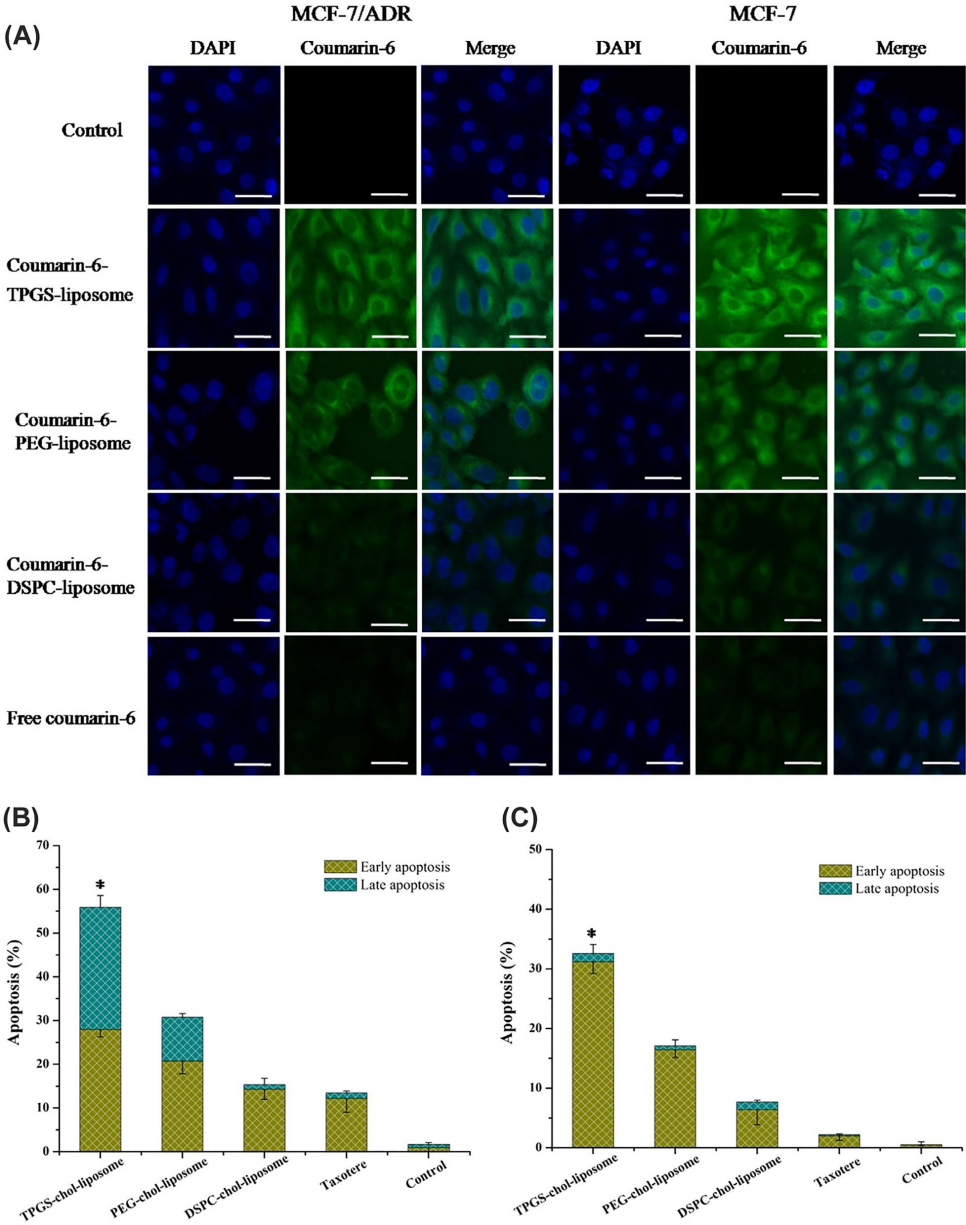
DTX-loaded NPs within liposomes. **A** Assessment of liposomal penetration into MCF-7 and MCF-7/ADR cells using confocal laser scanning microscopy (CLSM) after a 2-h exposure to different liposomal formulations, including free coumarin-6, TPGS-coumarin-6 liposome, PEG-coumarin-6 liposome, and DSPC-coumarin-6 liposome. The scale bar is set at 50 µm. The capacity of the various liposomal configurations to induce apoptosis in **B** MCF-7 cells and **C** MCF-7/ADR cells, with statistical significance denoted by *P < 0.05 when compared to TPGS-chol-liposome (n = 3) (Reprinted with permission from [89])

Li et al. [89] developed liposomes encapsulating DTX and coated with d-alpha-tocopheryl polyethylene glycol 1000 succinate (TPGS) were crafted as an innovative drug delivery mechanism to counteract multidrug resistance (MDR) and enhance the treatment of breast cancer. Demonstrated in Fig. 7A, liposomes modified with TPGS-chol significantly concentrated DTX within cancer cells. TPGS played a crucial role in boosting drug accumulation intracellularly by countering the overexpression of *P*-glycoprotein (p-gp). Consequently, assays for cell uptake and apoptosis revealed that TPGS-chol-liposomes markedly curtailed cell growth and overturned MDR in breast cancer cells known for their resistance (Fig. 7B). Moreover, liposomes with a TPGS coating offered superior protection for DTX from the reticuloendothelial system (RES) compared to those coated with PEG (also known as stealth liposomes) [89].

Kothari et al*.* [90] concurrently delivered DTX and alpha-lipoic acid (ALA) through SLNs as a method for treating breast cancer. NPs loaded with the combination of ALA and DTX showed significantly higher apoptosis and cytotoxicity compared to the free combination of ALA and DTX in 4T1 and MCF-7 breast cancer cells, which exhibited the increased efficiency of loaded SLNs with simultaneous use of drugs. As a result, developed SLNs increase cell uptake, apoptosis, and cytotoxic potential in breast cancer cells [90].

Maroufi et al. [91] prepared nanostructured lipid carriers (NLC) loaded with myricetin and co-delivery of DTX for treating breast cancer cells. NLCs loaded with myricetin decrease cell viability from 50 ± 2.3 to 40 ± 1.3% (p < 0.05). In addition, these DXT-loaded particles increased the percentage of apoptosis and the population of MDA-MB231 cells in subG1 arrest [91]. Another study by Zafar et al*.* [92] refined chitosan (CS)-grafted lipid nanocapsules (CLNCs) containing DTX and THQ, aimed at combating drug-resistant breast cancer. These optimized CLNCs, loaded with DTX and THQ, demonstrated enhanced controlled release and increased cytotoxicity towards MCF-7 and resistant TNBC cells. Results indicated that the synergistic delivery of DTX and THQ by CLNCs amplified the drugs' intracellular delivery, bolstered the anti-angiogenic response, and positioned them as viable options for the targeted treatment of both typical and resistant forms of breast cancer [92].

Naga et al*.* [93] reported that folic acid (FA)/PLGA polymer NPs were utilized to administer DTX to breast cancer cells. The inclusion of FA enables these NPs to specifically target breast cancer cells, thereby sparing healthy cells to a large extent. The MTT assay revealed that DTX encapsulated in FA/PLGA NPs exhibited markedly enhanced cytotoxicity. Furthermore, the presence of FA in the PLGA NPs notably obstructed the efflux of DTX by diminishing the expression of ABCG2 and MDR1 genes by 3.2 and 2.86 times, respectively, which are typically upregulated by free DTX. DTX within FA/PLGA NPs induced significant apoptotic activity, evidenced by the increased activation of caspase-9, caspase-3, and TP53 genes by 2.8, 1.6, and 1.86 times, respectively [93].

Choi et al. [94] investigated modifying the surface of PLGA NPs loaded with DTX by attaching Herceptin® (HCT) enhanced their uptake and cytotoxic effects on breast cancer cells. Through various methods such as adsorption (HCT-A-DTX- PLGA NPs), charged adsorption (HCT-C-DTX- PLGA NPs), and biosynthesis (HCT-B-DTX- PLGA NPs), DTX- PLGA NPs coated with HCT were effectively created. Among these, HCT-B-DTX- PLGA NPs demonstrated superior binding to breast cancer cells, along with enhanced stability, cellular uptake, and cytotoxicity compared to other DTX- PLGA NPs formulations in cell lines BT-474, SK-BR-3, and MCF-7 [94].

A liposomal formulation was engineered by Zhang et al. [95] to co-deliver dexamethasone (DEX) and DTX, with a designed sequential release mechanism aimed at modifying the tumor stroma to enhance drug penetration and accumulation within the tumor. This approach of sequential release generally extended the period before tumor degeneration and facilitated the distribution of DTX at the tumor site. Experimental results using tumor models indicated that the sequential dispensing of DEX and DTX from the liposomal co-delivery system exhibited superior anti-tumor efficacy and tumor inhibition rates, achieving up to 96.93% for the KB tumor, 86.03% for the KBv tumor, and 82.54% for the 4T1 tumor. Consequently, this combined nanomedicine approach is proposed as an effective strategy for enhancing the anti-tumor impact in breast cancer treatment [95].

Varshosaz et al. [96] designed copolymer micelles composed of poly(styrene-co-maleic acid) (SMA) and synthetic PEG (PAEEI-PEG) to deliver DTX to MCF-7 and MDA MB231 breast cancer cells. Experiments proved that micelles loaded with DTX increased the lifespan of animals, decreased tumor growth, and increased cytotoxicity up to 5 times compared to free DTX. As a result, SMA-PAEEI-PEG DTX micelles increased DTX cell absorption and apoptotic effect on breast cancer cells [96].

**Fig. 8:**
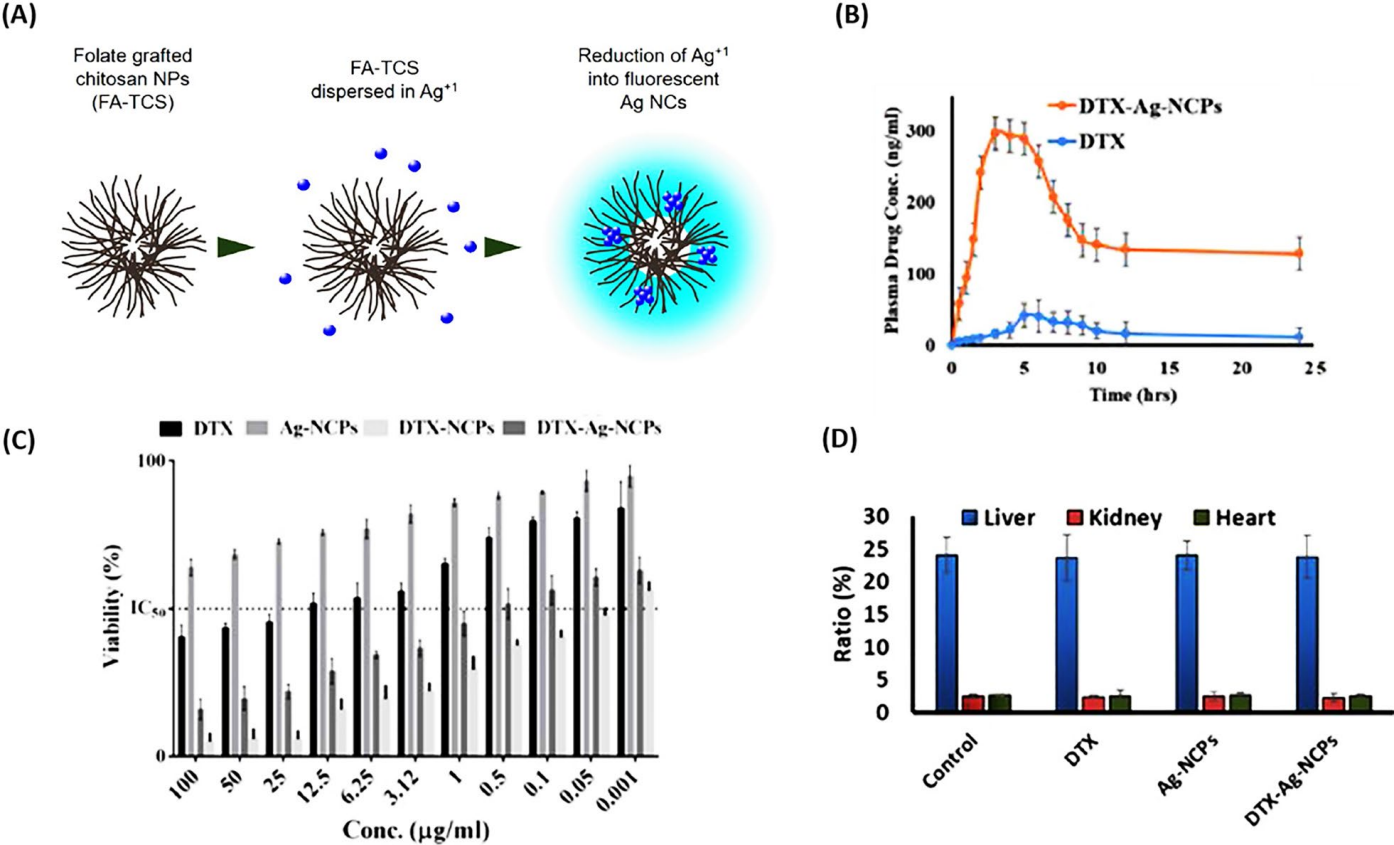
DTX within Silver Nanoclusters. **A** Creation of fluorescent AgNCs embedded in Chitosan. **B** The oral bioavailability of DTX suspension versus DTX-Ag-NCPs was evaluated in rabbits (n = 5) by measuring blood levels at set times (via HPLC) following the administration of 10 mg/kg of each formulation. **C** The comparative in vitro effectiveness of DTX suspension, DTX-Ag-NCPs, and Ag-NCPs on the human breast cancer cell line MDA-MB-231 was assessed. **D** The impact on the organ to body weight ratio in Swiss albino mice was examined following the OECD 425 guidelines for acute oral toxicity, with tests conducted on DTX, DTX-Ag-NCPs, and Ag-NCPs. The error bar denotes the Mean ± S.D. from three separate trials (Reprinted with permission from [97])

Sohail et al*.* [97] synthesized hybrid nanocapsules encapsulating DTX and silver nanoclusters (Ag NCs) (DTX-Ag-NCPs) were crafted using chitosan (CS) to enhance the oral bioavailability of DTX for the treatment of breast cancer (Fig. 8A). The DTX-Ag-NCPs notably extended the blood circulation half-life (~ 6.8-fold) and mean residence time (~ 6.7-fold) in comparison to a DTX suspension, demonstrating a significant increase in oral bioavailability, as shown in Fig. 8B (by ~ 9-fold). Figure 8C revealed that at lower concentrations, the DTX-Ag-NCPs, which incorporate both Ag and DTX, exhibited superior anticancer activity than NCs alone. Figure 8D confirmed the absence of significant 14-day acute oral toxicity in mice treated with DTX-Ag-NCPs, underscoring the safety and effectiveness of these nanoparticles in combating breast cancer [97].

Rarokar et al*.* [98] presented self-assembled nanocarriers (SANs) as a colloidal for DTX to control the release of anticancer drugs with improved stability. The obtained results showed a high amount of drug trapped in SANs loaded with DTX, and compared to the DTX-HA solution, which released the drug in 4 h, SANs released DTX in a controlled manner in 12 h. DTX-loaded SANs showed higher cytotoxicity in human breast adenocarcinoma (MDA-MB-231) cells. Therefore, optimizing and evaluating DTX-loaded SANs was an effective approach to the sustained release of anticancer drugs [98].

DTX micelles were created by Tan et al. [99] using mPEG-PLA and mPEG-PCL polymers for the purpose of breast cancer chemotherapy. Laboratory and animal studies indicated that these DTX micelles outperformed free DTX in halting tumor progression, with a significant decrease in systemic toxicity observed. Among them, DTX-mPEG-PCL micelles exhibited superior efficacy in suppressing tumor growth than DTX-mPEG-PLA micelles, likely owing to their stable accumulation in tumor tissues at elevated concentrations. Therefore, the physicochemical properties and antitumor effect of mPEG-polyester micelles make them promising agents for breast cancer chemotherapy [99].

Sahu et al*.* [100] created a nanosuspension that combines curcumin (CRM) with DTX to boost anti-breast cancer effects. They found that using CRM to inhibit p-gp alongside DTX significantly enhanced cytotoxicity in the MCF-7 cell line, outperforming individual drug suspensions, owing to improved cellular uptake in vitro. In vivo studies demonstrated an up to 70% increase in tumor suppression, attributed to heightened solubility, tumor cell sensitization, and p-gp inhibition in mice bearing MCF-7 tumors. Enhanced bioavailability and superior distribution contributed to the increased anticancer efficacy of CRM and DTX combination [100].

All these studies showed that encapsulation of DTX in various hydrogels and polymeric carriers have been used for breast cancer treatment, while majority of them remained in in vitro and/or in vivo phases and did not reach to the clinical trials. However, investigation on the applicability and efficacy of these DTX-encapsulated materials on the breast cancer therapy should be continued to determine the most efficient materials and nanoplatforms.

##### Lung cancer

**Fig. 9:**
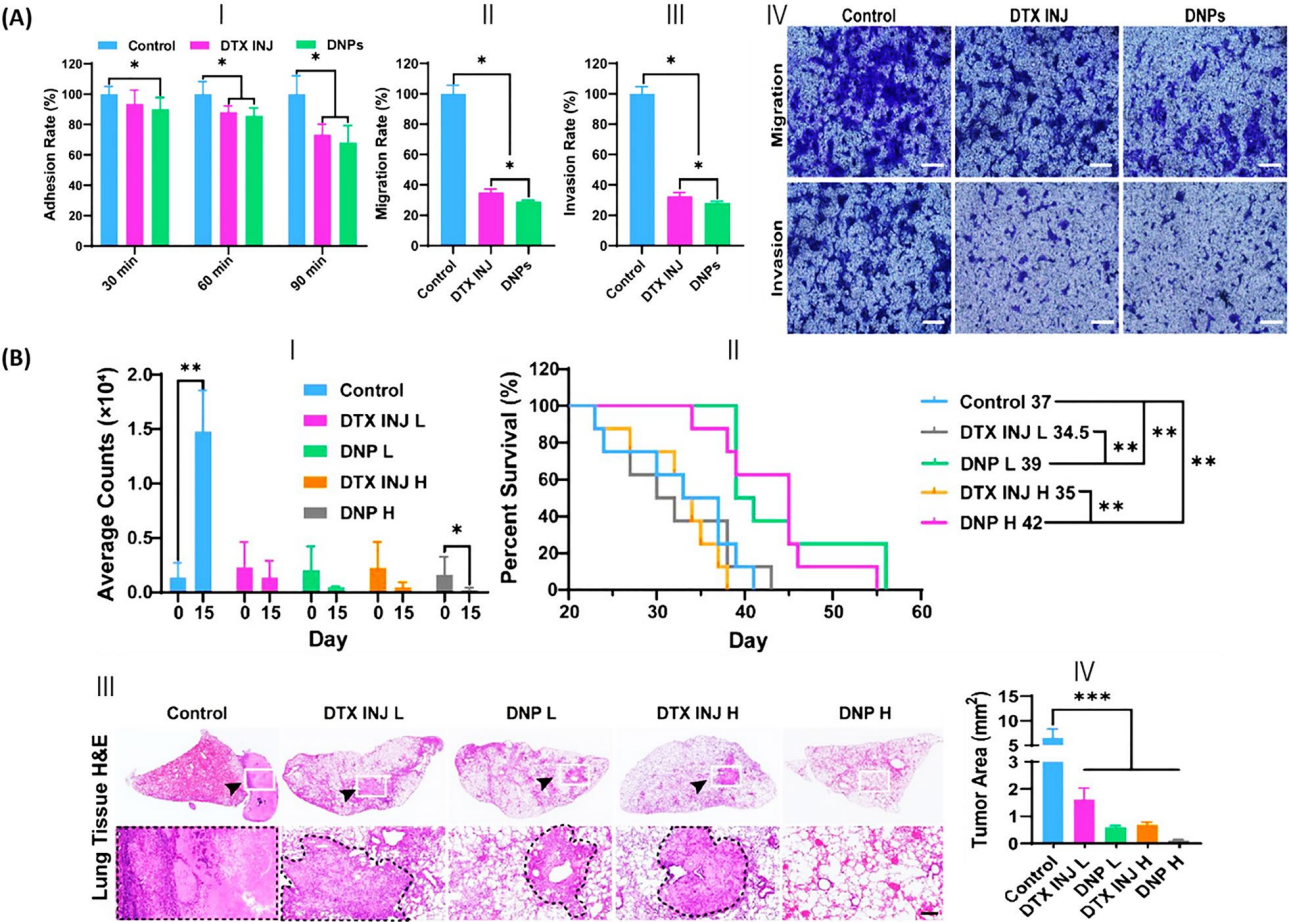
HSA nanoparticles loaded with DTX targeting non-small cell lung cancer. **A** Evaluation of DNP's effect against metastasis in vitro. (I) Assessment of PC9 cell adhesion following treatment with DNPs (DTX INJ) or DTX. (II) The rate of cell migration and (III) invasiveness derived from (IV). (IV) Photographic evidence of cells migrating or invading through matrigel-coated transwell barriers post-treatment with DNPs or DTX, stained with crystal violet and captured using a fluorescent microscope. Scale bar = 200 µm. **B** Assessment of DNP's antitumor activity in a live mouse model. (I) Tumor site count averages. (II) Survival rate monitoring of mice under treatment. (III) Quantification of lung H&E staining and tumor dimensions utilizing OLYMPUS OlyVIA software, with (IV) tumor areas highlighted by dotted black lines. Scale bar = 200 µm. Data represent means ± SD. Significance levels are indicated as *p < 0.05, **p < 0.01, and ***p < 0.001 between the groups indicated (Reprinted with permission from [101])

Along with breast cancer treatment, DTX encapsulation was used for lung cancer therapy. Studies showed that multiple researchers investigated several carriers to determine the efficacy of DTX-encapsulated materials in lung cancer therapy. In a previous study, human serum albumin (HSA)-loaded NPs, enhanced with medium-chain triglyceride (MCT) for stability through an adapted nanoparticle albumin-bound (Nab) technology, were engineered by Cheng et al*.* [101]. These NPs demonstrated superior absorption by NSCLC cells compared to DTX alone, leading to more effective inhibition of cell growth and adhesion (Fig. 9A-(a)), migration (depicted in Fig. 9A-(b), (d)), and invasion (illustrated in Fig. 9A-(c), (d)). Moreover, Fig. 9B-(a), (b) illustrate that DNPs possess superior inhibitory capabilities on both primary and metastatic tumor sites, also extending the average lifespan of mice with orthotopically implanted PC9-Luc tumors. Additionally, the outcomes of hematoxylin and eosin (H&E) staining presented in Fig. 9B-(c) reveal a decrease in lung tumor foci in mice treated with DNPs in comparison to those treated with DTX. However, systemic toxicity, organ toxicity, and blood toxicity were reduced compared to DTX injection. The results suggest that the developed DNPs, distinguished by their increased therapeutic efficacy and decreased toxicity, offer considerable potential for clinical application in the treatment of non-small cell lung cancer [101].

**Fig. 10:**
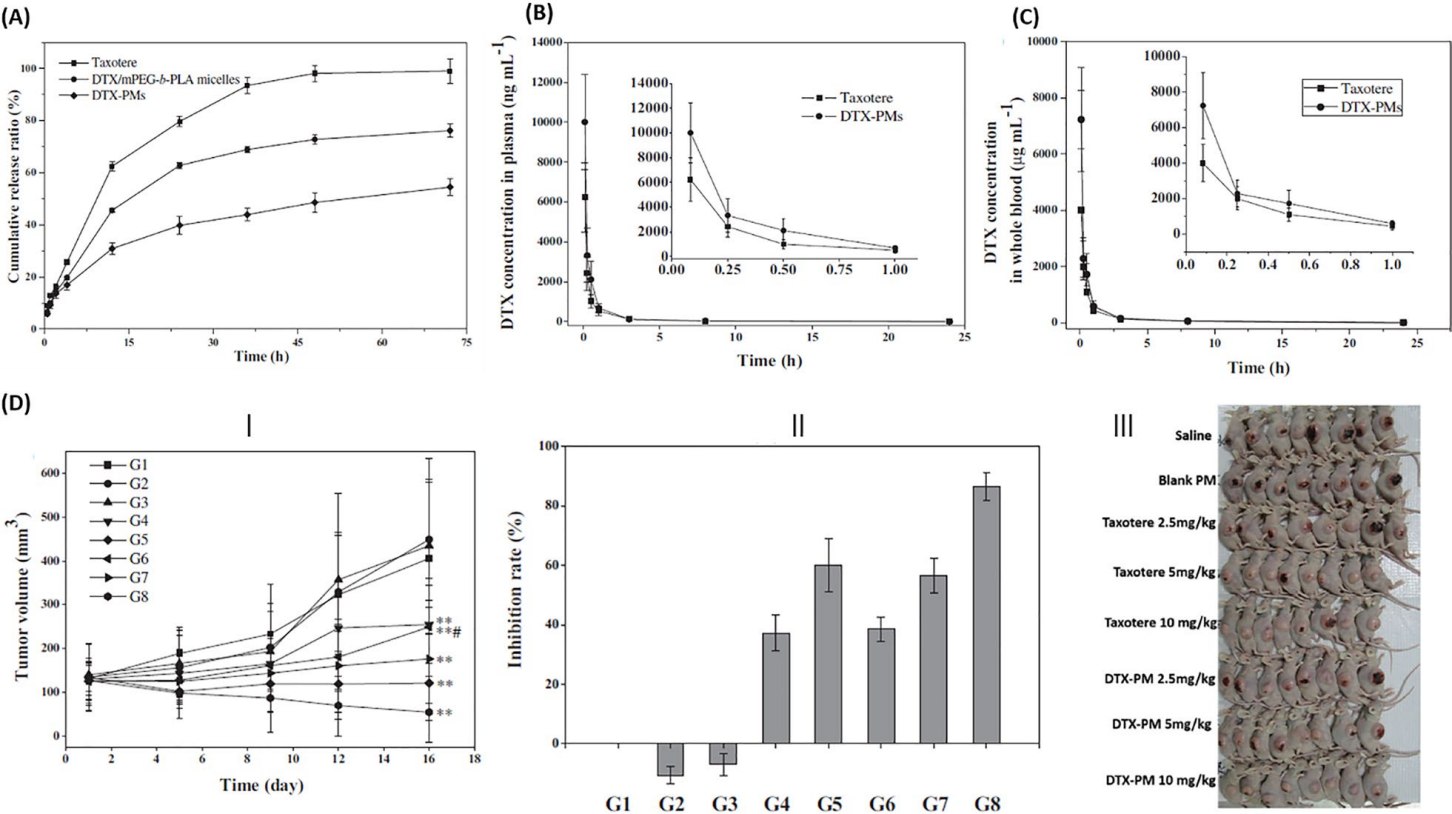
DTX-loaded mPEG-b-PLA-Phe(Boc) micelle studies. **A** Comparative release patterns of DTX from Taxotere®, DTX/mPEG-b-PLA micelles, and DTX-PMs. Examination of the distribution of Taxotere® and DTX-PMs in **B** the entire blood volume and **C** plasma of Sprague–Dawley rats. **D** Assessment of the A549 xenograft model’s reaction to treatment in vivo, including (I) a graph tracking tumor growth over time, (II) analysis of the tumor suppression rate, and (III) photographs of mice bearing A549 tumors (Reprinted with permission from [102])

Gong et al. [102] encapsulated DTX in mPEG-b-PLA-Phe(Boc) micelles (DTX-PMs) to improve its stability and antitumor efficacy in lung cancer. Compared to Taxotere, DTX-PM was a tumor-targeted and sustained-release formulation with higher drug potency, which made it more useful for convenient transportation and clinical use (Fig. 10A). Pharmacokinetic study indicated that an elevated concentration of DTX in both blood and plasma, attributed to the enhanced stability of the micellar formulation (Fig. 10B and C). Hence, DTX-PMs improved the accumulation of DTX and inhibited tumor growth in human NSCLC (A549) tumor-bearing Balb/c nude mice (Fig. 10D) [102].

Moreover, targeted DTX liposomes (DTX-LPs) were developed by Mengia et al. [103] to study drug metabolism in rabbits, rats, and mice as a treatment for NSCLC. The findings indicated a negligible metabolic distinction between DTX-LPs and DTX injections (DTX-IN), as demonstrated in rabbit feces, which did not result in any notable metabolic delay. Moreover, the liposomal administration of DTX enhanced its concentration in the lungs, limited its distribution in non-target tissues, and heightened its anticancer efficacy. This characteristic augmented the success of DTX treatment and diminished adverse effects in NSCLC therapy [103].

Cadete et al*.* [104] developed nanocapsules composed of hyaluronic acid (HA) loaded with DTX (DTX-HA-NCs) for the treatment of lung cancer through an uncomplicated self-assembly method. The results showed that nanocapsules with low cytotoxicity improved the intracellular delivery of the drug and increased the efficiency of DTX encapsulation and stability in plasma. Therefore, by loading DTX, the nanocapsules were effectively absorbed by A549 lung cancer cells and inhibited cancer cell growth [104].

**Fig. 11:**
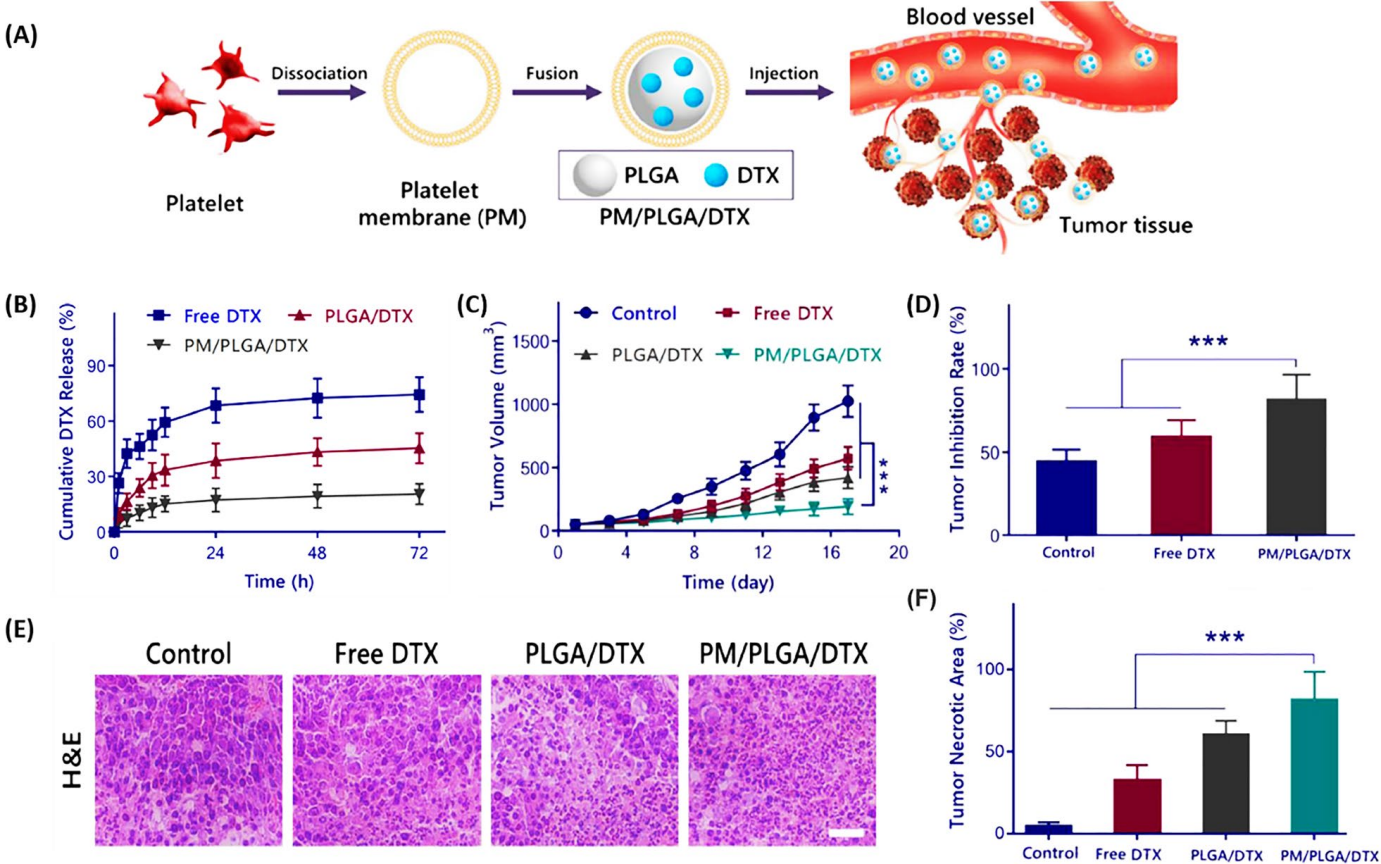
Biomimetic nanoparticles encapsulating DTX for lung cancer treatment. **A** Schematic representation of in vivo delivery using PM/PLGA/DTX nanoparticles. **B** Graph showing the release of DTX in vitro in PBS (pH 7.4) at 37 °C for PLGA/DTX, PM/PLGA/DTX, and free DTX, with averages shown as mean ± SD (n = 3). **C** The volume of tumors in mice and **D** the rates of tumor suppression following administration of free DTX, PLGA/DTX, and PM/PLGA/DTX, reported as mean ± SD (n = 8), ***P < 0.001. **E** Examination of tissue pathology; H&E-stained tumor tissue images from treatments with free DTX, PLGA/DTX, and PM/PLGA/DTX. Scale bar = 50 μm. **F** Quantitative assessment of the necrotic areas within tumors for each treatment group (Reprinted with permission from [105])

Chi et al*.* [105] developed a novel nanoplatform based on DTX-loaded PLGA nanoparticles coated with platelet membrane (PM) (PM/PLGA/DTX) for targeted therapy of lung cancer (Fig. 11A). In vitro, the PM/PLGA/DTX nanoplatform inhibited tumor cell growth and reduced DTX release (Fig. 11B). The immune evasion and cancer-targeting abilities of PM allowed PM/PLGA/DTX to prolong blood circulation and effectively target lung cancer. Figure 11C and D illustrate the successful targeting and concentration of PM/PLGA/DTX within tumors, which substantially reduces the viability of A549 lung cancer cells in a live model. Moreover, the systemic toxicity of DTX, depicted in Fig. 11E, is markedly diminished when administered as PM/PLGA/DTX, in contrast to its free form. Therefore, this platform provided an effective strategy for treating lung cancer without causing severe systemic toxicity [105].

**Fig. 12:**
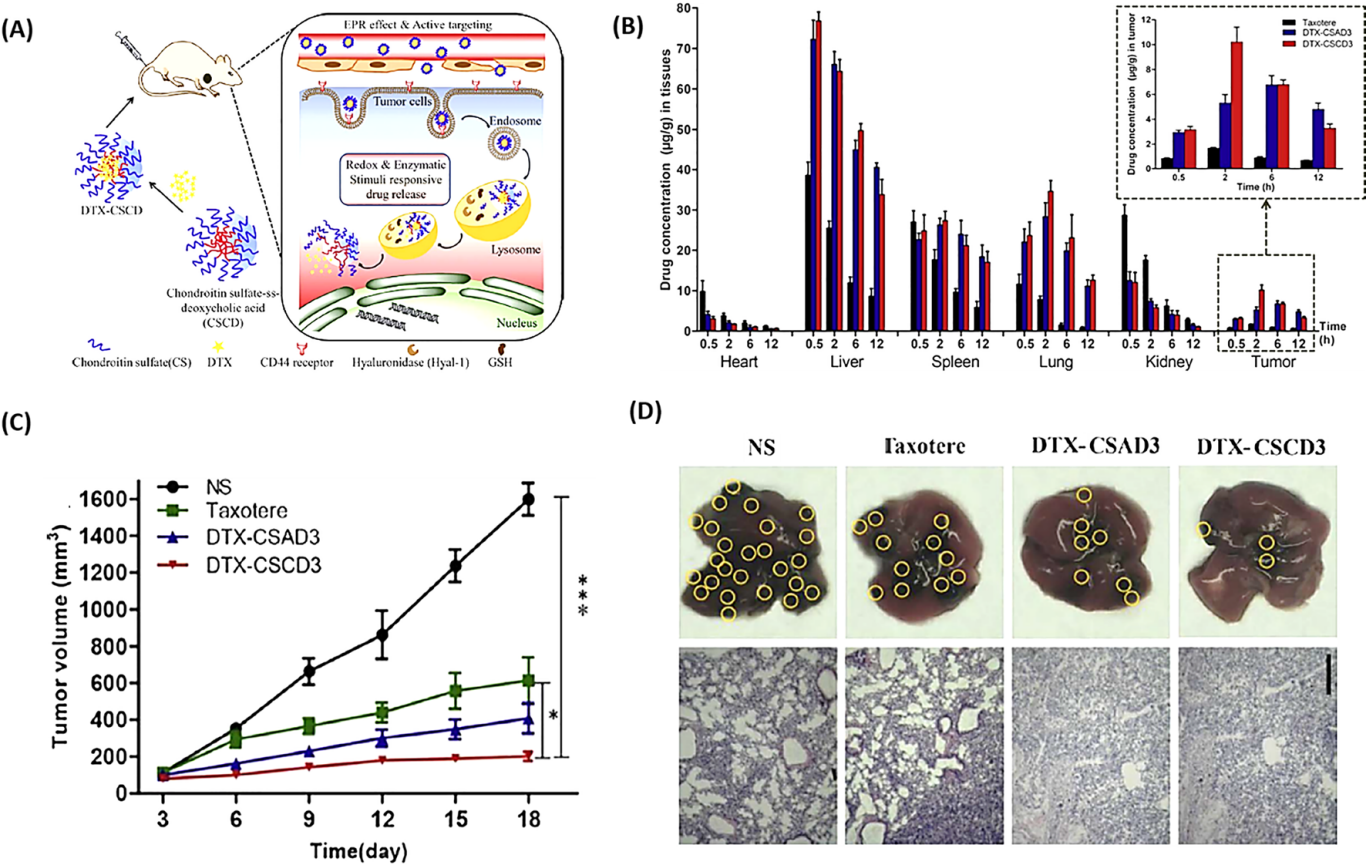
Nanoparticles delivering DTX for melanoma metastasis and growth suppression. **A** DTX-CSCD nanoparticles self-assemble, target tumors, and dispense medication in reaction to redox/enzymatic triggers. **B** Live tracking of DTX distribution in melanoma-bearing mice post intravenous delivery via the tail with Taxotere®, DTX-CSAD3, and DTX-CSCD3, averaged data as mean ± SD (n = 3). **C** Comparative anti-tumor effectiveness of normal saline (NS), Taxotere®, DTX-CSAD3, and DTX-CSCD3 nanoparticles in mice with B16F10 tumors, results expressed as mean ± SD (n = 5), with statistical significance indicated by *p < 0.05, ***p < 0.001, compared to the DTX-CSCD3 group. **D** Examination of lung tissue via H&E staining from B16F10 melanoma mice treated with various compounds, where yellow circles highlight areas of lung metastasis (Reprinted with permission from [106])

Liu et al. [106] synthesized DTX-loaded smart enzyme/redox responsive chondroitin sulfate self-assembled nanoparticles (DTX-CS SANs) for melanoma treatment (Fig. 12A). As shown in Fig. 12B, compared to Taxotere, DTX-CS SANs improved the distribution of DTX in tumors and lungs [about 4.4-fold the value of the area under the curve (AUC)]. Therefore, these NPs reduced in situ tumor volume (Fig. 12C) and lung metastatic (Fig. 12D) formation through DTX-induced apoptosis and inhibition of metastasis-induced protein expression. The rapid release and accumulation of drugs in tumor tissues through these NPs provided hope for the treatment of melanoma [106].

##### Prostate cancer

Prostate cancer ranks as the second most frequently diagnosed cancer in men, following lung cancer. [72]. When prostate cancer advances to a metastatic stage, treatment options include chemotherapy and androgen deprivation therapy. DTX plays a role in combating prostate cancer by exerting its anticancer effects through inhibiting microtubule depolymerization, and countering bcl-2 and bcl-xL gene resistance effects. The primary mode of action for DTX in prostate cancer treatment is its ability to stabilize microtubules by binding to tubulin. This binding disrupts the typical mitotic processes and induces cell cycle arrest in the G and M phases, leading to cell death through apoptosis due to static polymerization [107].

**Fig. 13:**
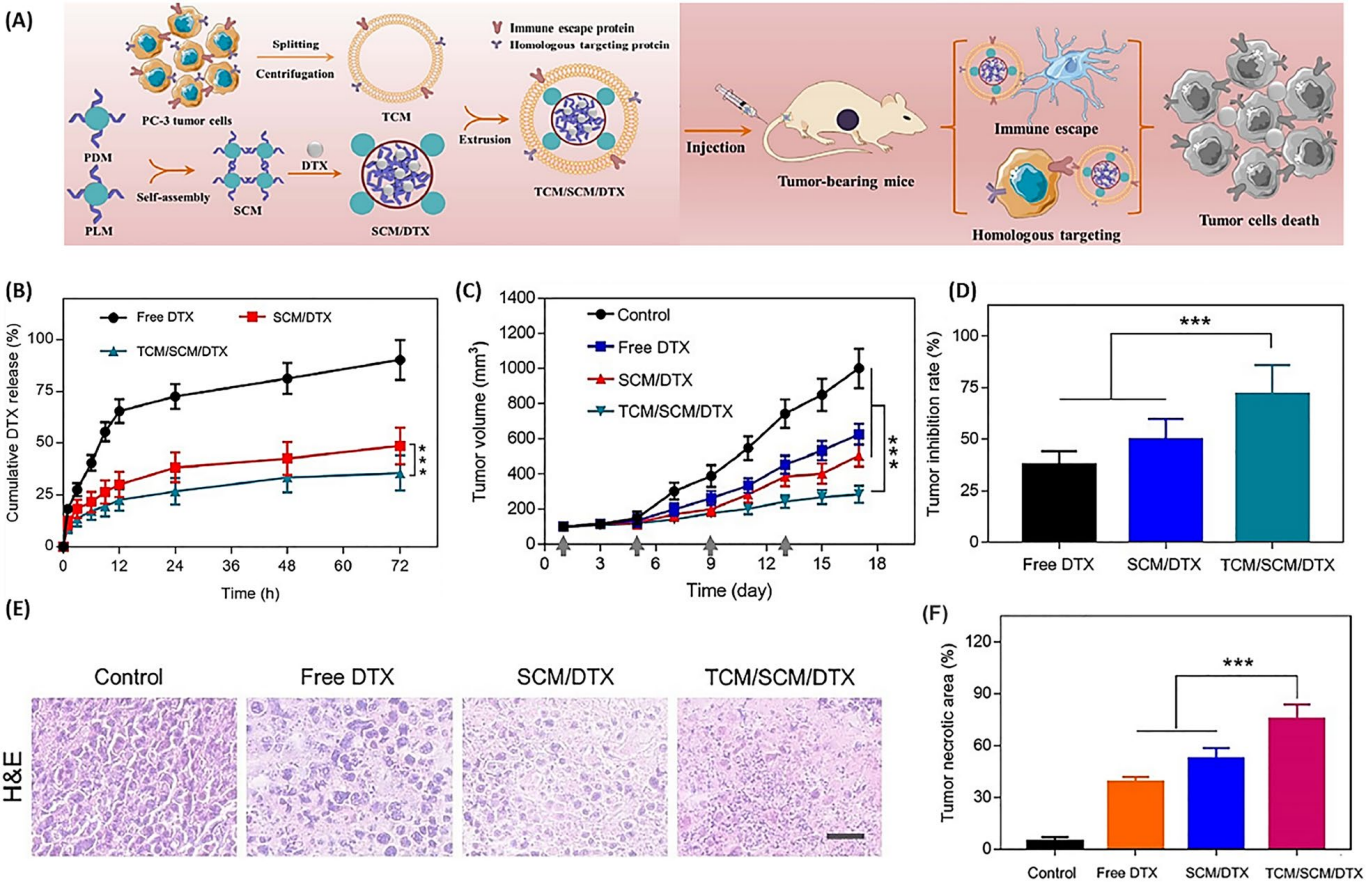
Biomimetic nanoparticles mirroring cancer cells for improved prostate cancer treatment. **A** Illustration of a biomimetic platform enveloped by a cancer cell membrane, enhancing immune evasion and targeted delivery of TCM/SCM/DTX analogs for better antitumor effects. **B** Release profile of DTX in vitro from TCM/SCM/DTX and SCM/DTX at pH 7.4 and 37 °C, with results shown as mean ± SD (n = 3). Observations of antitumor activity in a living organism. **C** Alterations in tumor size, **D** tumor suppression rates, and **E** microscopic examination of tumor tissue sections stained with H&E in mice treated with DTX, SCM/DTX, and TCM/SCM/DTX (Reprinted with permission from [108])

Hong et al. [108] designed a nanoplatform with an outer shell of a homotypic tumor cell membrane (TCM) and an inner core of a stereo complex micelle (SCM) loaded with the drug DTX for targeted prostate tumor therapy (Fig. 13A). As shown in Fig. 13B, TCM coating reduced the release and sustained release of DTX during the bloodstream. Both in vitro and in vivo experiments demonstrated that TCM/SCM/DTX nanocarriers accumulate in tumor tissue, reducing tumor volume (Fig. 13C) while increasing growth inhibition (Fig. 13D). Furthermore, Fig. 13E and F proved that this nanoplatform significantly reduces the toxicity of DTX in tissues and organs [108].

**Fig. 14:**
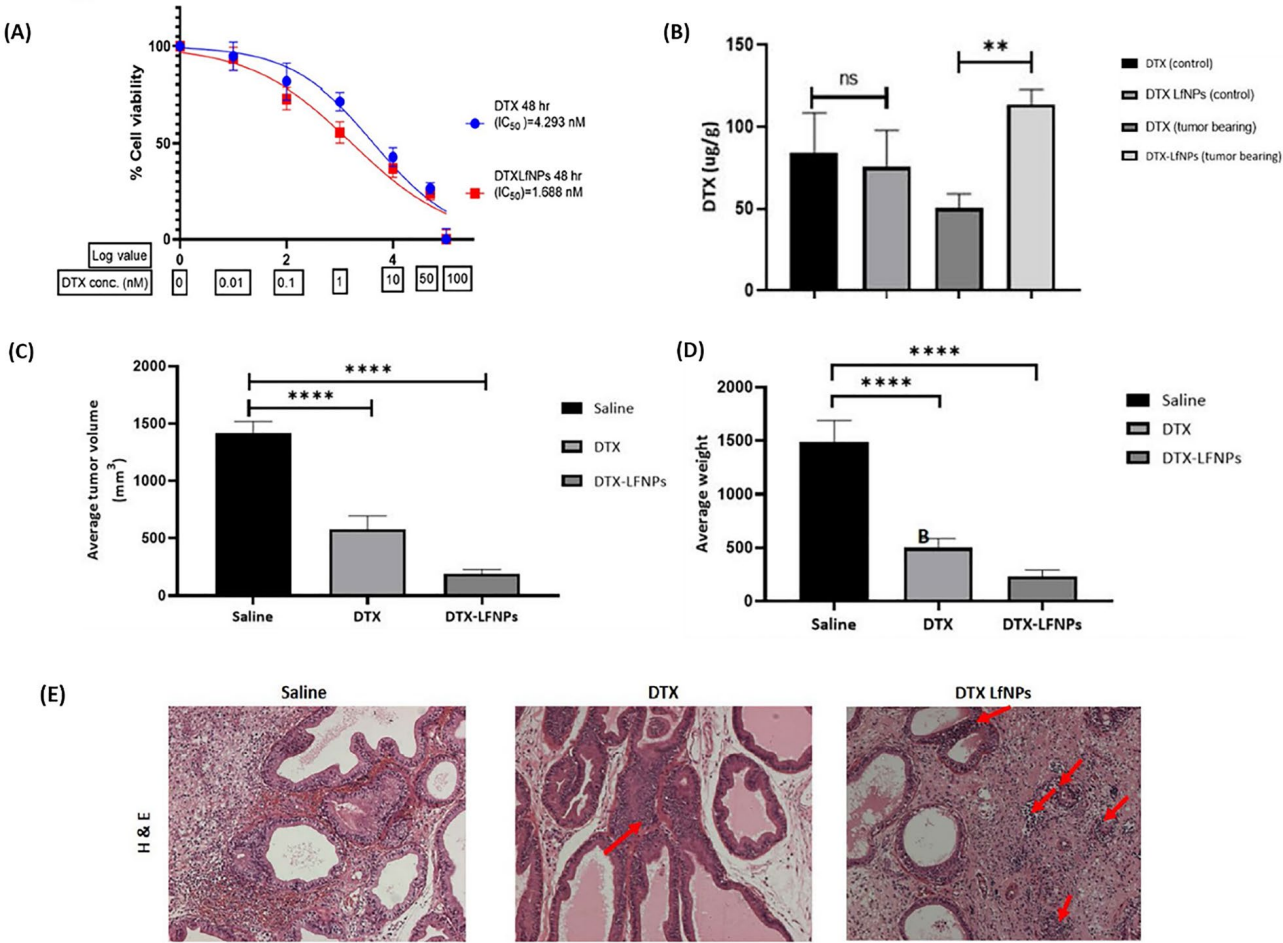
Enhanced efficacy and safety of DTX in prostate cancer treatment through the synergistic use of lactoferrin. **A** Observations on how DTX and DTX-LfNPs impact the growth of Mat-LyLu prostate cancer cells over 48 h. Following 48 h post-administration, a reduction in the IC50 value for DTXLfNPs was noted in comparison to that of soluble DTX. **B** The bioavailability of DTX within the prostate cancer tissues of male Wistar rats over a 24-h period. The results are presented as mean ± SD for six subjects, with **P < 0.01 (using Student’s t-test). **C** Evaluation of tumor suppression: Measurements of tumor volumes and (**D**) weights were taken at the three-week mark. The data are displayed as mean ± SD for six subjects, and ****P < 0.0001 (according to ANOVA’s post-test). **E** Histological examination of prostate tissues treated with either DTX or DTX-LfNPs at 20× magnification, using saline as a baseline control. Areas of necrosis are marked with red arrows (Reprinted with permission from [109])

Muj et al. [109] loaded lactoferrin (Lf) nanoparticles with DTX (DTX-LfNPs) and reported that both DTX and Lf existed in biologically active forms and could inhibit processes related to tumor metastasis and prostate cancer chemotherapy. Based on their report, DTX-LfNPs increased the anti-proliferative activity (2.5 times) (Fig. 14A) and the bioavailability of the drug in the prostate (2 times) compared to free DTX (Fig. 14B). Examining the effectiveness of DTX-LfNPs in an orthotopic prostate cancer caused by Mat-Ly-Lu cells in a mouse model showed increased anticancer activity in terms of prostate tissue volume (Fig. 14C) and weight (Fig. 14D) regression compared to DTX. Figure 14E shows that DTX-LfNPs outperform free DTX in cancer tissue treatment effectiveness [109].

Li et al. [110] designed targeted nanoparticles (NPs) with co-delivery of DTX and doxorubicin (DOX) (DDC-NPs) to achieve maximal anti-prostate cancer effects with minimal side effects. The results showed that DDC-NPs increased drug accumulation in tumors and decreased non-specific accumulation in normal organs. Therefore, due to the synergistic effect of drugs, DDC-NPs improved the therapeutic effect and reduced toxicity in vivo, which could be a potential perspective in the clinical chemotherapy of PCA [110].

**Fig. 15:**
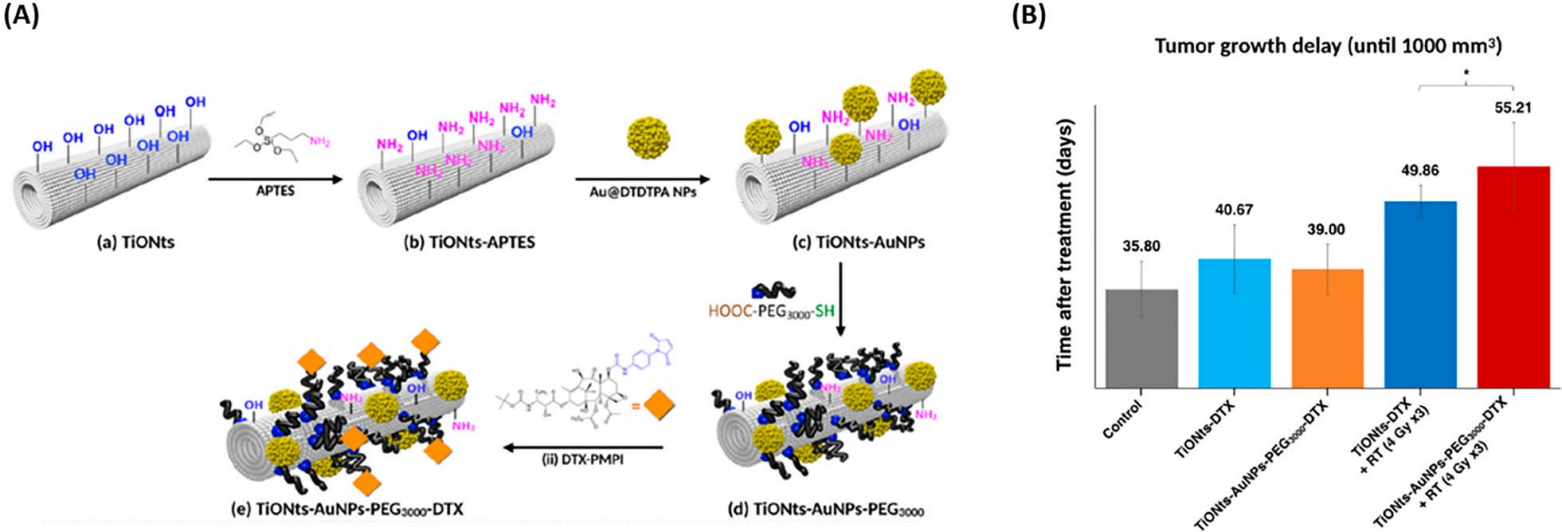
Titanate Nanotubes Engineered with Gold Nanoparticles DTX. **A** Illustration of TiONts-AuNPs-PEG3000-DTX. **B** The therapeutic effect of TiONts-DTX and TiONts-AuNPs-PEG3000-DTX injection into PC-3 xenografted tumors with or without radiotherapy administered in three daily doses of 4 Gy in three groups of 6–7 animals each. * p = 0.035, the nonparametric Mann–Whitney test was utilized to perform the analysis (Reprinted with permission from [111])

Loiseau et al. [111] synthesized nanohybrids based on titanate nanotubes (TiONts) engineered with gold NPs and loaded with DTX (TiONts-AuNPs-PEG3000-DTX) for radiotherapy enhancement in the treatment of xenografted prostate tumors (Fig. 15A). The DTX loading in the synthesized nanohybrids increased the cytotoxic activity on PC-3 human prostate adenocarcinoma cells. Furthermore, the TiONts helped to keep the AuNPs inside the tumor, delay tumor growth, and improve treatment efficiency (Fig. 15B) [111].

Polymeric NPs made of PLGA were used to encapsulate both abiraterone acetate (AbrA) and DTX (DTX) by Sokol et al. [112] offering a combined approach of hormone therapy and chemotherapy for treating prostate cancer. The study revealed that such NPs, loaded with AbrA and DTX, demonstrated greater cytotoxic effects and were more effectively internalized by human prostate adenocarcinoma cells than the individual drugs in their free states, thanks to the mechanism of synergistic interaction. [112].

Su et al. [113] reported that a lecithin-stabilized micellar drug delivery system was developed to encapsulate DTX, aiming to minimize systemic toxicity while maximizing treatment efficacy for prostate cancer. They reported that DTX encapsulated within developed drug delivery system demonstrated superior tumor growth inhibition, decreased toxicity, and increased antitumor effects on DU145 prostate cancer cell lines. Live studies indicated that this system carrying DTX enhanced the drug's concentration at the tumor location, allowing for an injectable drug dose that was 2–2.5 times greater than that of the unencapsulated drug. As a result, this drug delivery system can be used as a nanocarrier capable of high drug dosage to improve the effectiveness of chemotherapy [113].

**Fig. 16:**
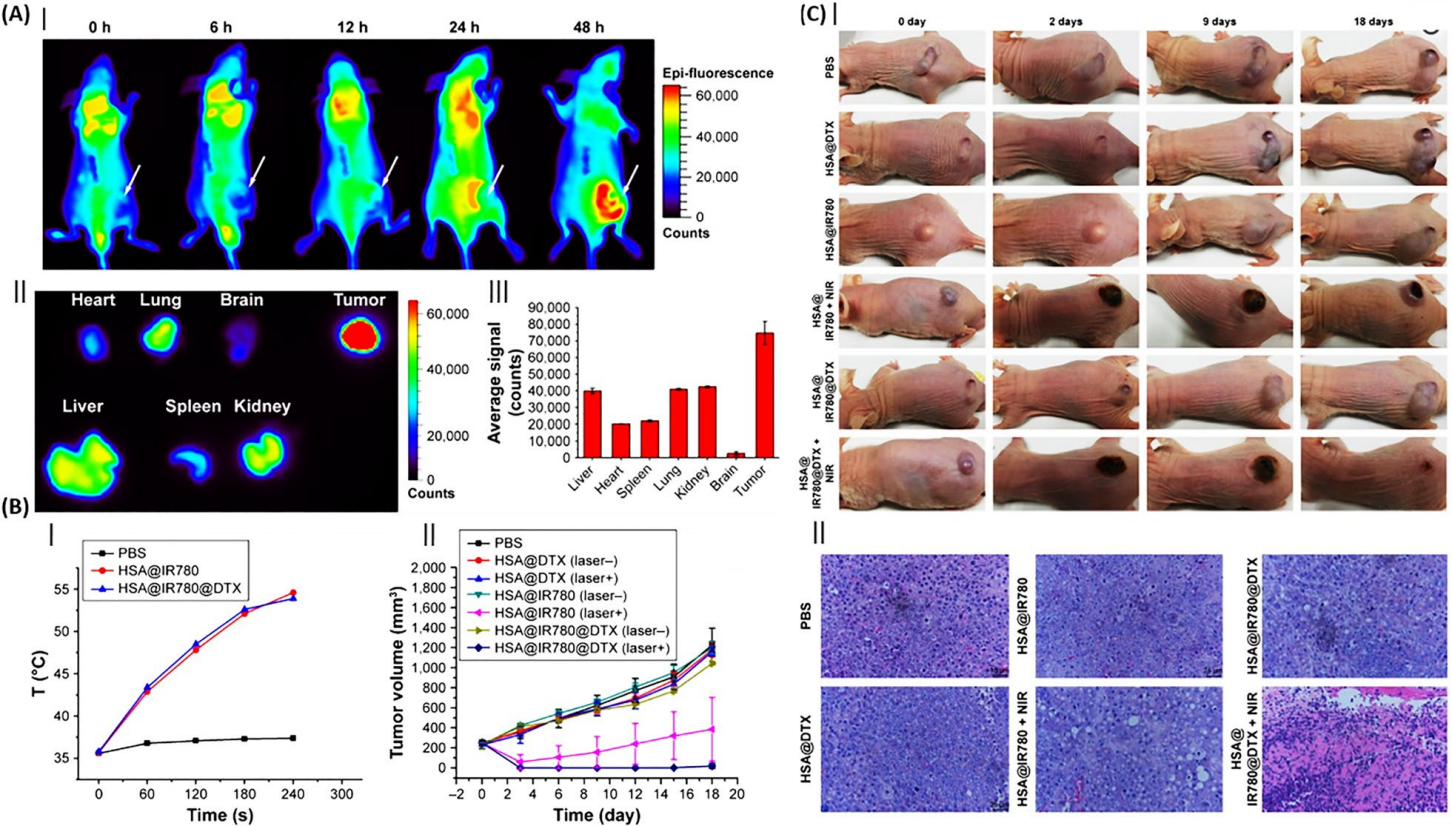
Nanoparticles of albumin for combined therapeutic strategies in prostate cancer. **A** Near-infrared (NIR) fluorescence imaging of prostate cancer in mice using HSA@IR780@DTX nanoparticles. (I) Fluorescence imagery following nanoparticle injection into prostate cancer-afflicted mice, with tumor locations marked by white arrows. (II) Fluorescence imagery of dissected major organs from mice 48 h post-injection. (III) Semiquantitative analysis of nanoparticle distribution across major organs, presented as mean ± SD (n = 3). **B** Employing a combination of PTT and chemotherapy in a subcutaneous tumor model. (I) Comparative analysis of thermal response in tumor-bearing mice treated with PBS, HSA@IR780 nanoparticles, and HSA@IR780@DTX nanoparticles, with results shown as mean ± SD (n = 3). (II) Tumor growth trajectories in different mouse cohorts subjected to varied treatments, with error bars denoting standard errors of the mean (n = 5). **C** In vivo assessment of nanoparticle therapeutic impact. (I) Comparative imagery of mice with prostate cancer pre- and post-treatment. (II) Microscopic examination of tumor tissues stained with H&E following nanoparticle treatment (Reprinted with permission from [114])

Lian et al. [114] reported that multifunctional NPs were created by integrating IR780 (a near-infrared dye) and DTX with human serum albumin (HSA) to form HSA@IR780@DTX. This composite was designed for precise imaging and a dual therapeutic approach combining photothermal therapy (PTT) and PDT for treating castration-resistant prostate cancer. Tumor-bearing mice administered with HSA@IR780@DTX NPs underwent near-infrared fluorescence imaging showed that these particles tended to accumulate in the tumor over time, which might be due to increased permeability and retention effects (Fig. 16A). Also, Fig. 16B demonstrated that the simultaneous effect of DTX and the use of laser increased the inhibition of the growth of xenografted prostate tumors in mice, indicating the combined effect of PTT/PDT with chemotherapy. Additionally, the combination of HSA@IR780@DTX and NIR laser severely destroyed tumor cells (Fig. 16C) in mice during in vivo therapeutic testing [114].

#### Techniques used for encapsulating docetaxel in nanoparticles

NPs enhance the solubility, bioavailability, and targeted release of DTX [115]. The nano drug delivery systems employed for DTX encompass polymeric nanoparticles, liposomes, micelles, liquid formulations, and inorganic nanoparticles. Nanoparticle's size, surface charge, and wettability affect the pharmacokinetics, solubility, blood circulation time, and biocompatibility of DTX, which has been employed in the management of multiple types of cancer [115]. NPs are attached to DTX is integrated into NPs either non-covalently, by encapsulation near their surface, or covalently, through the creation of a prodrug [116, 117]. Key considerations for selecting the optimal nanoparticle for DTX encompass its high solubility under physiological conditions stability in the cancer cell environment, controlled slow release, and minimal side effects on surrounding cells [118]. DTX can be incorporated into NPs during synthesis, coating, physical absorption, physio-sorption, and chemisorption [119].

##### Liposomes

Liposomes consist of multiple phospholipid bilayers that encapsulate an aqueous core. They can be categorized based on the number of phospholipid layers into two types: monolayer liposomes with a single bilayer and multilayer liposomes with multiple bilayers [120]. In the liposome structure, water-soluble drugs are trapped in the aqueous compartment, and lipophilic drugs are trapped between two phospholipid layers [121]. The use of liposomes as DTX carriers leads to increased drug transfer efficiency, drug protection in harsh environments, reduced systemic toxicity, reduced interaction with plasma proteins, reduced phagocytosis, and increased blood circulation time of DTX compared to other nanoparticle systems [122]. Kamoun et al. [18] employed a liposome linked to an Ephrin receptor A2 antibody (notably overexpressed in diverse tumors) to transport DTX. In their study with animal models (including mice, rats, and dogs), they demonstrated that the liposomal formulation ensures a stable and sustained release of the drug, decreases the active dose circulating in the bloodstream, lowers hematotoxicity, and enhances both the tumor penetration and the antitumor efficacy of DTX.

Li et al. [103] developed DTX-loaded targeted liposomes for the treatment of non-small cell lung cancer. Their results showed that liposomes delayed the metabolism of DTX and increased its accumulation in the lungs in animal models of rabbits, rats, and mice, which indicated that this formulation is safe for clinical use. Li et al. [89] used DTX liposomes coated with D-alpha-tocopheryl polyethylene glycol 1000 succinate (TPGS) to reverse multidrug resistance in breast cancer and compared the results with PEG-coated liposomes. Their results showed that DTX was well encapsulated in liposomes, and led to its stable and continuous release. In addition, TPGS coating inhibited the expression of P-gp and increased the accumulation of DTX in MCF 7/ADR cells, leading to an increase in its efficiency.

##### Polymer nanoparticles

Polymeric NPs, both solid and colloidal, made from biodegradable polymers, enhance DTX delivery by ensuring high bioavailability, efficient encapsulation, controlled release, and extended blood circulation while evading the reticuloendothelial system [122–124]. These cost-effective NPs accumulate in tumors due to prolonged circulation, exploiting irregular vasculature and the enhanced permeation and retention effect, enabling diverse cancer treatments through encapsulation, core/shell models, and DTX conjugation [125, 126]. These inexpensive and accessible nanoparticles through encapsulation, core/shell model, and DTX conjugation have led to a wide range of cancer treatments [127, 128].

In addition, using biomimetic coatings, peptides, antibodies, and small molecules on the surface of these particles can help improve their performance, stability, circulation time, and accumulation in tissues with damaged vessels [127]. Generally, drug release from these nanoparticles is done by swelling, bulk erosion, and diffusion [121]. Ungaro et al. [129] developed polycaprolactone (PCL) and PEO core/shell NPs for passive DTX delivery, with hydrated PEO in the corona and PCL in the core, trapping the drug. Their results showed that the use of PEO-PCL NPs as a DTX carrier reduced red blood cell hemolysis, inhibited the growth of breast and prostate cancer cells, and reduced toxicity in animal models.

Zhao et al. [130] used polymer nanoparticles consisting of phase change material (core), polypyrrole, and hyaluronic acid (shell) as DTX carriers. Their results demonstrated that these NPs, with their photoacoustic effects and temperature-dependent drug release, aid in the precise positioning of the drug, enabling photothermal targeting of tumor cells and inhibiting tumor growth in 4T1 mice. Furthermore, these NPs proved effective in photoacoustic imaging, ensuring accurate tumor localization and penetration into tumor cells. Xu et al. [131] incorporated a combination of DTX and cimetidine into chitosan polymer nanoparticles and showed that these nanoparticles improved the oral bioavailability, water solubility, and absorption of DTX formulation in the small intestine.

##### Inorganic nanoparticles

Inorganic nanoparticles such as manganese oxide [132], gold [133], mesoporous silica [134], mesoporous polydopamine [135], and quantum dots [52] have also been considered as DTX carriers due to their unique properties. For example, the favorable properties of gold NPs, which have made it a favorable option for drug release and cancer diagnosis, include the ability to bind to various biological compounds, high biocompatibility, electro-optical properties (related to the surface plasmon band), adjustable size and shape, and ease of surface modification and functionalization (generally with thiolate and laminate) [136]. Folic acid has also been considered due to its interaction with the folate receptors of cancer cells [136].

Due to its expansive surface area, considerable porosity, robust mechanical and chemical stability, and suitable biocompatibility, mesoporous silica, characterized by its hollow interior, has gained recognition [137]. Mesoporous polydopamine is also one of the other NPs that have received attention due to its biodegradability, compatibility with different tissues, favorable magnetic and electro-optical properties in cancer treatment, and the release of chemotherapy drugs [135]. Using magnesium oxide as a DTX carrier, Abbasi et al*.* [132] showed that this combination reduces the required dose and increases the efficiency of chemotherapy with DTX.

Thambiraj et al. [133] used gold NPs in DTX formulation, then by functionalizing this complex with thiol-PEG-amine and connecting it to folic acid, they were able to make it a targeted tool for prostate cancer treatment. Their results showed that this complex has resulted in damage to cancer cells and the death of 40%. Muthu et al. [52] utilized a blend of quantum dot NPs and DTX, functionalized with d-alpha-tocopheryl polyethylene glycol 1000 succinate mono-ester, for targeting MCF-7 breast cancer cells. They stated that the quantum dot improved the targeted drug release and the imaging capability of cancer cells.

**Fig. 17:**
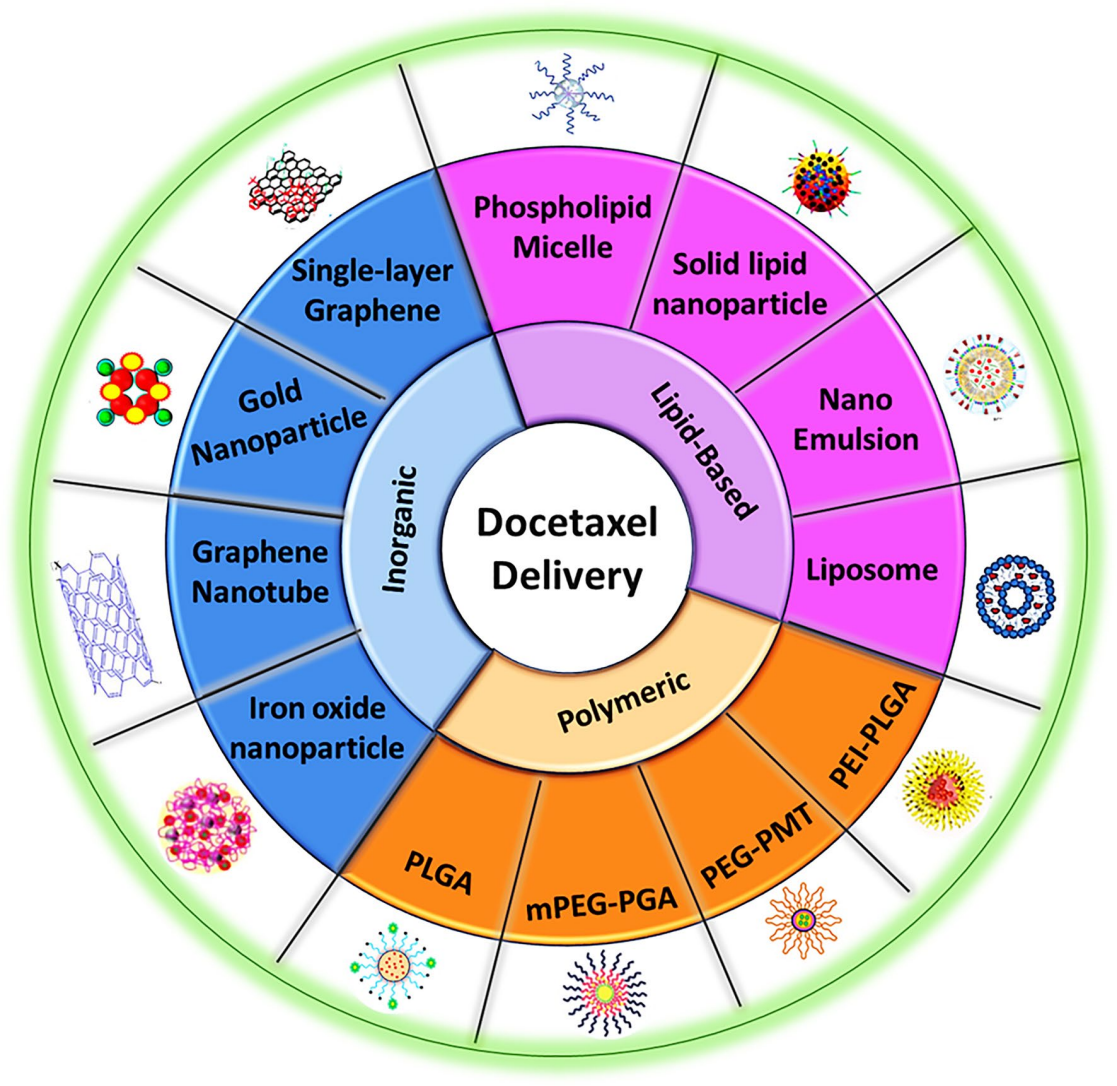
Schematic illustration of various nanoparticle-based DTX delivery systems

After discussing the majority of utilized carriers for DTX delivery, it could be seen that various polymeric and inorganic NPs, as well as various polymeric NFs have been used in DTX encapsulation, which could be efficient in cancer therapy, targeted drug delivery, cellular regeneration, and regenerative medicine approaches. In order to summarize these findings, all these nanoparticles are categorized in Fig. 17.

## Functionalization of nanocarriers for targeted release of docetaxel

The presence of specific receptors on tumor cells’ surfaces has led to the strategy of combining nanoplatforms with targeted functional groups or ligands specific to tumor tissue. This approach actively directs DTX to the tumor, enhancing the effectiveness of nanoplatforms in cancer therapy. Targeting agents used for DTX are monoclonal antibodies [138], transferrin [139], peptides [140], fatty acids [141], and folate [142].

The high selectivity of monoclonal antibodies in recognizing and binding to specific proteins on the surface of cancer cells helps to improve DTX efficiency and reduce side effects [143]. For example, the EGFR receptor is involved in the malignancy and growth of most tumors, including breast, stomach, and ovary. This receptor is involved in stimulating metastasis, angiogenesis, proliferation, and death prevention of cancer cells [144]. Eloy et al. [145] used liposomes (containing DTX) conjugated to cetuximab (as an anti-EGFR antibody) to treat prostate cancer. Their results showed that this targeting increased toxicity, cell uptake, and efficiency of liposomes. Patel et al. [146] also showed that PLGA NPs containing DTX and conjugated to cetuximab increased DTX efficacy in reducing tumor proliferation and growth of non-small cell lung cancer.

Considering the high affinity of cancer cells for some peptides such as vascular endothelial growth factor (VEGF), EGF, Asn-Gly-Arg, and RGD, it is possible to connect them to nanoplatforms containing DTX for improved targeting efficiency. Chen et al. [147] used RGD-modified micelles as DTX carriers in breast cancer treatment. Their results indicate a continuous release system to increase drug absorption in tumor cells, reduce side effects, and improve tumor activity.

Transferrin is a membrane protein with a high expression in various cancer cells such as the brain, breast, and lung [148]. This homodimer protein consists of glycosylated subunits connected by two disulfide bonds. Gan et al*.* [127] conjugated transferrin to poly(lactide)-d-α-Tocopheryl PEG succinate NPs containing DTX to help brain cancer treatment by crossing the blood–brain barrier. Their results showed that the transferrin improved the blood–brain barrier crossing, cell uptake, and toxicity of cancer cells (229% more than Taxotere).

By attaching folate ligands to nanoplatforms, which target the abundantly expressed folate receptors in many cancer types, the precision of DTX release can be enhanced. Werner et al. [149] conjugated folate on polymer-lipid NPs carrier of DTX so that these NPs absorption (as an effective radiosensitizer) is increased in the head and neck tumor cells in a time-dependent manner. The attachment of polyunsaturated fatty acids to nanoplatforms leverages the tumor cells' high consumption of these acids driven by their energy demands for proliferation to enhance the delivery and efficacy of DTX in targeting tumor cells while minimizing its side effects [150].

## Challenges of docetaxel encapsulation

DTX delivery systems offer numerous advantages over conventional DTX formulations in terms of enhancing therapeutic efficacy, minimizing toxicity, and reducing dose-dependent side effects. However, it is important to acknowledge that each delivery system comes with its own set of limitations.

There are two fundamental types of bonding mechanisms between the nanocarrier and docetaxel: non-covalent and covalent. In both cases, key properties are essential for effective drug delivery, including proper solubility under physiological conditions, stability until reaching target cancer cells, selectivity in targeting tumor cells, slow release within tumor cells, and minimal side effects on healthy cells. Some nanocarriers are also engineered with targeting agents to enhance specificity.

The non-covalent attachment approach faces challenges primarily related to effective encapsulation by nanocarriers and maintaining stability in route to tumor cells [151]. Conversely, the covalent approach requires prodrugs to remain stable in the bloodstream, convert efficiently to their active form, and release the drug upon reaching cancer cells. The decision on which nanocarrier to utilize depends on the specific target and addressing existing limitations.

For instance, SLNs as a drug delivery system have limitations such as rapid elimination by the reticular endothelial system and low drug loading efficiency due to their compact lipid crystal lattice. This can restrict the amount of drug available for cellular uptake. Moreover, challenges in dissolving drug molecules in lipid matrices used for SLNs further complicate the drug-loading process. Furthermore, nanoparticle systems like SLNs, polymeric micelles (PMs), and lipid-polymer hybrid NPs (LPHNPs) offer the advantage of a hydrophilic surface, facilitating prolonged circulation in the bloodstream and allowing for active and passive targeting [152].

Although active and passive targeting strategies have shown improved efficacy for DTX delivery, they primarily focus on enhancing drug delivery to the target site to enhance cancer cell uptake. Therefore, selecting the appropriate nanocarrier for treating different cancers hinges on understanding their specific roles and capabilities in drug delivery optimization. The advantages and disadvantages of nanoformulations have been mentioned in Table 2.

**Table 2:** Advantages and limitations of various nanocarriers

Types of nanocarriers	Advantages	Disadvantages
Nano fibers	Simple equipment	Limited to specific polymers, toxic solvents, not scalable
Polymeric nanoparticles	Biodegradable, biocompatible, targeted drug delivery, low toxicity	Low loading capacity
Liposomes	Loaded both hydrophilic and hydrophobic drugs, easily fuse with cell membrane, low toxicity, biocompatible	Low capacity, instability
Solid-lipid nanoparticles	Good tolerability, ease to scale-up, low cost, high physical stability	Drug expulsion, unpredictable agglomeration, premature release
Gold nanoparticles	Easy to modify, can be prepared in broad size range	Optical signal not strong, toxicity, tumor targeting efficiency low

## Clinical trials of docetaxel in various drug delivery systems

DTX continues to be utilized in cancer treatment owing to its therapeutic efficacy and the formulation's medicinal properties [153]. As mentioned in the previous sections, a wide range of in vitro and in vivo studies have shown the therapeutic potential of different DTX drug delivery systems for cancer diseases. In this regard, multiple clinical trials have been conducted on new formulas based on DTX, as shown in Table 3.

**Table 3:** Clinical trials of DTX-loaded NPs

Trial identifier	Formulation	Cancer	No. of Human volunteers	Parameters evaluated	Phase	Status
NCT01300533	BIND-014, a DTX-containing nanoparticle targeting PSMA	Metastatic cancer Cancer solid Tumors	52	MTD, PK	1	Completed
NCT02479178		Urothelial Carcinom Cholangiocarcinoma Cervical cancer Squamous cell Carcinoma of head and neck	73	Objective response rate (ORR)	2	Terminated
NCT02283320		Non-small cell lung carcinoma Squamous cell lung carcinoma	69	Disease control rate (DSR), progression-free survival, overall survival (OS), duration of response (DOR), time to response, safety and tolerability and ORR	2	Completed
NCT01792479		Non-small cell lung cancer (NSCLC)	64	ORR, safety and tolerability of BIND-014	2	Completed
NCT01812746		Metastatic castration-resistant prostate cancer (mCRPC) Prostate cancer	42	Radiographic progression-free survival (rPFS), safety and tolerability of BIND-014	2	Completed
NCT01151384	Liposome encapsulated DTX (LE-DT)	Solid tumors	30	MTD, PK and anti-tumor effect	1	Completed
NCT01186731		Metastatic pancreatic cancer	40	Response rate of tumor size reduction	2	Completed
NCT01103791	DTX-PNP	Advanced solid malignancies	19	MTD, dose limiting toxicity (DLT), PK, ORR	1	Completed
NCT03712423	89 Zirconium Cripec DTX PET	Solid tumor	7	Detection (visual and quantitative) of [89Zr]-Df-CriPec® DTX in tumour lesions	1	Completed
NCT03742713	DTX containing CriPec® nanoparticles	Cancer Ovarian cancer	25	ORR, progression free survival (PFS), DOR, Time to progression (TTP), DCR, safety and tolerability	2	Completed
NCT02442531	CriPec® DTX (DTX containing nanoparticles)	Cancer metastatic Cancer solid tumors	33	Safety and tolerability, MTD and PKs	1	Completed
NCT01041235	ATI-1123 is a liposomal formulation of DTX	Solid tumor breast cancer ovarian cancer pancreatic cancer non-small cell lung	29	Safety, tolerability, PKs, and antitumor activity	1	Completed
NCT03671044	Nanosomal DTX Lipid Suspension	Triple negative breast cancer	657	ORR, PFS and OS	3	Recruiting

mPEG-DEPE: 1,2-Distearoyl-*sn*-glycero-3- phosphoethanolamine-*N*-methoxy-poly(ethylene glycol 2000) (mPEGDSPE); PAEEI-PEG: poly (amide-ether-ester-imide)-polyethylene glycol; mPEG-PCL: monomethoxy poly(ethylene glycol)-poly(ε-caprolactone); TMSP: tumor microenvironment-sensitive polypeptides; mPEG-b-PLA-Phe(Boc): *N*-(tert-butoxycarbonyl)-l-phenylalanine endcapped methoxy-poly(ethylene glycol)-block-poly(d,l-lactide)

Regarding the assessment of neutropenia in novel formulation of DTX, it should be noted that neutropenia is defined as an abnormally low number of neutrophils, which are crucial white blood cells for combating infections [154]. DTX, like many chemotherapeutic agents, targets rapidly dividing cells, including both malignant cells and healthy bone marrow cells responsible for producing neutrophils. This action leads to a reduction in neutrophil counts, increasing the patient’s susceptibility to infections [155]. Neutropenia is frequently reported as a dose-limiting toxicity in patients undergoing DTX treatment [156]. Studies have indicated that up to 75–80% of patients treated with DTX experience neutropenia, which can vary in severity depending on the dosage and regimen [157]. Here we compared each clinical trials and research via neutropenia aspects.

BIND-014 is a nanoparticle encapsulating DTX and a hydrophobic PEG crown decorated with small molecule prostate-specific membrane antigen (PSMA) targeting ligands. Clinical trials have been conducted using these particles to treat various cancers. In phase I clinical trials, BIND-014 demonstrated increased antitumor activity compared to conventional DTX [158]. These NPs were effective in metastatic castration-resistant prostate cancer and NSCLC but did not show any effect in cervical and head and neck cancer. However, phase II of the study was suspended due to low response rates and other manufacturer-related factors [159], and the BIND company was acquired by Pfizer [160].

NeoPharm, Inc. has developed a novel proprietary DTX delivery system named LE-DT, which was employed in phase I (NCT01151384) and II (NCT01186731) clinical trials for the treatment of patients with solid malignant tumors and pancreatic cancer. Findings from the phase I study indicated a reduction in side effects and the potential for administering a higher dose to enhance LE-DT's efficacy. Moreover, DTX, as part of protein-bound taxane therapy, demonstrated promising efficacy in the treatment of pancreatic cancer. The application of this drug system in phase I resulted in clinical benefits (stable disease + partial response) for 41% of the patients [161].

This phase I study of liposome encapsulated DTX (LE-DT) in patients with solid tumors (NCT01151384) found that neutropenia was among the dose-limiting toxicities (DLTs) observed. The trial aimed to determine the maximum tolerated dose (MTD) and evaluate the pharmacokinetics and antitumor effects of LE-DT. The study reported manageable toxicity profiles, with neutropenia being a significant concern at higher doses.

In the NCT01103791 study, polymeric NPs containing DTX (PNP-DTX) were injected in different doses from 20 to 75 mg/m^2^ into patients with advanced solid malignancies. This study aimed to determine the maximum tolerated dose (MTD) and the recommended dose of DTX-PNP. The results showed that DTX-PNP administration up to a dose of 60 mg/m^2^ every 3 weeks was well tolerated by patients who were heavily treated [162].

Recently, in a clinical study (NCT03712423), a DTX-entrapping polymer nanoparticle (89Zr-CPC634) was labeled, and the biodistribution and tumor accumulation in patients with solid tumors were investigated through positron emission tomography/computed tomography (PET/CT) imaging. PET/CT imaging results showed tumor accumulation in different doses of CPC634 during treatment [163]. CPC634 is a novel system with DTX entrapped inside CriPec-stabilized NPs.

The initial phase one clinical trial, NCT02442531, aimed to determine the maximum tolerable dose (MTD) of CriPec DTX for treating patients with solid tumors. This study’s goals were to evaluate CriPec DTX's safety, tolerability, pharmacokinetics (PKs), and pharmacodynamics and to establish the MTD. Researchers noted that neutropenia was identified as a significant adverse event, which influenced the determination of the MTD. The study’s findings indicated that while CriPec DTX could be safely administered, high doses led to cumulative and reversible neutropenia.

It was found that CPC634 could be safely administered; however, at higher doses, reversible and cumulative skin toxicity was noted. Additionally, a phase II study (NCT03742713) was designed to examine CPC634's efficacy and intratumoral penetration in individuals with platinum-resistant ovarian cancer. The findings indicated that while treatment with CPC634 was feasible, it did not demonstrate significant clinical activity in individuals with recurrent platinum-resistant ovarian cancer. The primary side effects observed were gastrointestinal in nature [164].

This study utilized CPC634, which involves DTX encapsulated in CriPec NPs, intended to boost drug accumulation in tumors through targeted release at the site, thereby enhancing therapeutic outcomes. The objective was to explore the impact of CPC634 on treating patients with advanced epithelial ovarian cancer who had shown resistance to prior platinum-based chemotherapy treatments. Despite the potential for enhanced tumor drug accumulation, the presence of neutropenia posed challenges for patient management and dose optimization. The findings indicated that while administering CPC634 was feasible, it did not demonstrate significant clinical effectiveness in patients with recurrent platinum-resistant ovarian cancer [165].

ATI-1123 employs a liposomal delivery system that integrates DTX into the liposome's lipid bilayer using protein-stabilized nanoparticles (PSN). A phase I clinical trial was undertaken to assess the potential clinical application of ATI-1123 in treating patients with advanced solid tumors. The findings revealed that the drug was well-tolerated, exhibited a favorable pharmacokinetic (PK) profile, and demonstrated encouraging antitumor efficacy. In addition, due to liposomal encapsulation, hypersensitivity reactions were generally reduced (NCT01041235) [166]. Nanosomal DTX lipid suspension (NDLS) consists of DTX particles suspended in a lipid-based formulation. The study emphasized the need for careful monitoring and supportive interventions to manage neutropenia in treated patients.

Since 2013, Intas Pharmaceuticals Ltd. has been supplying this drug under the brand name DOCEAQUALIP 20/80 in India. In a multicenter, observational, retrospective analysis (NCT03671044), the treatment records of women across India who received breast cancer therapy with NDLS-based chemotherapy (75–100 mg/m^2^; every three weeks) were examined. Investigations showed that chemotherapy based on NDLS was effective and tolerable in the treatment of breast cancer [167]. This phase I study examined another novel DTX delivery system such nanosomal DTX lipid suspension for solid tumors. Neutropenia emerged as a significant adverse event, influencing dose adjustments and supportive care strategies. The trial results reinforced the commonality of neutropenia across different DTX formulations and the importance of managing this side effect to maintain treatment efficacy and patient safety.

Also, the NCT01812746 study evaluated the effects of a DTX-based treatment in this formulation DTX-containing nanoparticle targeting PSMA. Neutropenia was one of the primary hematologic toxicities observed. The trial results underscored the need for supportive care measures to manage neutropenia in patients undergoing treatment.

The NCT02479178 study investigated the use of BIND-014, a DTX-containing nanoparticle targeting PSMA, for DTX in non-small cell lung cancer. Neutropenia was a noted adverse effect, consistent with the known side effects of DTX, which is often associated with significant hematologic toxicities including neutropenia.

Moreover, in the NCT02283320 trial, which focused on a DTX-containing nanoparticle targeting PSMA for solid tumors, neutropenia was a prominent side effect. The study aimed to assess the safety and tolerability of the new formulation and found neutropenia to be a critical adverse event, requiring close monitoring and dose adjustments. Also, the NCT01792479 trial explored DTX in a different delivery system for solid tumors by a DTX-containing nanoparticle targeting PSMA. Neutropenia was again a major adverse effect, highlighting the commonality of this side effect across various DTX-based treatments. The trial aimed to optimize dosing to balance efficacy and safety, with neutropenia being a key factor in dose-limiting toxicity assessments. Overall, Neutropenia remains a common and critical adverse event across various clinical trials involving different formulations of DTX. These studies highlight the need for effective management strategies to mitigate this side effect and optimize therapeutic outcomes for patients.

## Future perspectives

DTX continues to establish itself as a formidable chemotherapeutic agent against a wide array of human cancers. The integration of nanotechnology into chemotherapy protocols holds the promise of transforming DTX delivery through improved water solubility, targeted tumor cell selection, controlled drug release, optimized pharmacokinetics, and diminished side effects. Over the last ten years, there have been considerable advancements in utilizing nanoplatforms for DTX delivery, highlighting the promising potential of this strategy as elaborated in this review.

Confronting DTX’s limitations, forward-looking formulation designs are homing in on targeted delivery mechanisms. Engineering the perfect nanocarrier is a complex endeavor, demanding precise drug-to-carrier ratios for maximal combined effects and grappling with the issue of inconsistent drug loading, which adds to the complexity of nanocarrier development. Presently, the conventional DTX formulations are hindered by limited efficiency in targeting cancer cells directly, which leads to substantial adverse effects and necessitates dosage limitations to safeguard healthy tissue. The inherent toxicity of DTX poses significant risks to normal cell populations.

To attain therapeutic objectives, there is a need for increased attention on the antitumor effectiveness and biodistribution of these agents, assessed through both in vitro and in vivo studies. While nanotechnology-based delivery systems offer significant benefits, the journey towards their clinical adoption is fraught with obstacles, including safety concerns, complex manufacturing processes, and rigorous quality control measures [168]. With no FDA-approved nanotech-based DTX formulations to date, it is crucial to navigate past these hurdles through rigorous research and innovation. Therefore, the clinical and commercial adoption of DTX-loaded nano formulations hinges on our ability to address these critical issues, paving the way for breakthroughs that could redefine cancer treatment paradigms in regenerative medicine.

## Conclusions

In conclusion, merging DTX with nanotechnology-based delivery mechanisms, notably nanofibers and nanoparticles, marks a notable progress in the domains, from oncology to regenerative medicine. This review has systematically examined the structure, sources, and derivatives of DTX, alongside its mechanism of action and bioavailability, laying the groundwork for understanding its therapeutic potential. The development of DTX-loaded nanoplatforms has been highlighted, showcasing their applications in effectively targeting and treating cancerous tissues with reduced toxicity and enhanced efficacy. The encapsulation techniques utilized for integrating DTX into nanofibers and nanoparticles have been explored, revealing the innovative approaches to improving drug delivery. Furthermore, the biological and physical effects of incorporating DTX into these nanoplatforms have been discussed, underscoring the benefits of targeted therapy and controlled release in cancer treatment.

The significant impact of these nanoplatforms in regenerative medicine, particularly through the facilitation of tissue engineering and the promotion of cellular regeneration, points to the versatile applications of DTX beyond conventional chemotherapy. As we move forward, the insights garnered from current research and clinical applications offer a promising outlook for the future of cancer therapy and regenerative treatments. It is imperative that future perspectives focus on overcoming the remaining challenges, optimizing the therapeutic window of DTX, and further innovating delivery systems to maximize efficacy and minimize side effects. Continued research and development in this arena are crucial for harnessing the full potential of DTX-loaded nanoplatforms, ultimately leading to more effective, safer, and personalized treatment options for patients worldwide.

## Data Availability

All the data that support the findings of this study are available in this manuscript.
